# Symmetric arrangement of mitochondria:plasma membrane contacts between adjacent photoreceptor cells regulated by Opa1

**DOI:** 10.1073/pnas.2000304117

**Published:** 2020-06-22

**Authors:** Ingrid P. Meschede, Nicholas C. Ovenden, Miguel C. Seabra, Clare E. Futter, Marcela Votruba, Michael E. Cheetham, Thomas Burgoyne

**Affiliations:** ^a^UCL Institute of Ophthalmology, University College London, EC1V 9EL London, United Kingdom;; ^b^Department of Mathematics, University College London, WC1E 6BT London, United Kingdom;; ^c^Centro de Estudos de Doenças Crónicas, Universidade Nova de Lisboa, 1169-056 Lisbon, Portugal;; ^d^School of Optometry and Vision Sciences, Cardiff University, CF24 4HQ Cardiff, United Kingdom;; ^e^Cardiff Eye Unit, University Hospital Wales, CF14 4XW Cardiff, United Kingdom

**Keywords:** rod photoreceptors, mitochondria, contact sites, tethers, dominant optic atrophy

## Abstract

Mitochondria are known to play an essential role in photoreceptor function and survival that enables normal vision. Within photoreceptors, mitochondria are elongated and extend most of the inner-segment length, where they supply energy for protein synthesis and the phototransduction machinery in the outer segment, as well as acting as a calcium store. Here, we examined the arrangement of the mitochondria within the inner segment in detail using three-dimensional (3D) electron microscopy techniques and show they are tethered to the plasma membrane in a highly specialized arrangement. Remarkably, mitochondria and their cristae openings align with those of neighboring inner segments. The pathway by which photoreceptors meet their high energy demands is not fully understood. We propose this to be a mechanism to share metabolites and assist in maintaining homeostasis across the photoreceptor cell layer. In the extracellular space between photoreceptors, Müller glial processes were identified. Due to the often close proximity to the inner-segment mitochondria, they may, too, play a role in the inner-segment mitochondrial arrangement as well as metabolite shuttling. OPA1 is an important factor in mitochondrial homeostasis, including cristae remodeling; therefore, we examined the photoreceptors of a heterozygous *Opa1* knockout mouse model. The cristae structure in the *Opa1*^*+/−*^ photoreceptors was not greatly affected, but the mitochondria were enlarged and had reduced alignment to neighboring inner-segment mitochondria. This indicates the importance of key regulators in maintaining this specialized photoreceptor mitochondrial arrangement.

## Significance

Mitochondria play an essential role in the homeostasis of the highly energy-demanding photoreceptors and enable normal vision. We used 3D electron microscopy to show that the mitochondria in the photoreceptor inner segment are tethered to the plasma membrane in a highly specialized arrangement. This includes mitochondria running alongside each other in neighboring inner segments, with evidence of alignment of the cristae openings. The cristae structure was not greatly affected in photoreceptors of a heterozygous *Opa1* knockout mouse model, but the mitochondria were enlarged and had reduced alignment to neighboring inner-segment mitochondria. This leads us to propose that mitochondria are arranged to share metabolites and assist in maintaining homeostasis across the photoreceptor cell layer.

Vertebrate photoreceptors are specialized neurons that provide vision by transducing light into electrical signals. The combination of phototransduction, neurotransmitter utilization, protein synthesis and transport, and repolarization after depolarization makes the energy consumption of photoreceptors greater than any other cell type in the body (1, 2). As a consequence, failure to fulfill their energy requirements often results in visual problems, including blindness. These include diseases such as Leber’s hereditary optic neuropathy, dominant optic atrophy (DOA), and Leigh syndrome that lead to malformed or dysfunctional mitochondria (3, 4). Most of the mitochondria of photoreceptors are housed within the inner segment (IS) region and are typically elongated, running along the long axis of the photoreceptor. Within the IS, mitochondria are well situated for uptake of extracellular metabolites via channels on the IS plasma membrane (PM) and can provide the necessary energy (in addition to aerobic glycolysis) for protein synthesis and for the phototransduction machinery of the adjoined outer segment (OS) (5). Furthermore, photoreceptor mitochondria have been shown to act as a Ca^2+^ buffer (6, 7). Ca^2+^ regulation is crucial for signaling, including phototransduction, membrane excitability, energy metabolism, cytoskeletal dynamics, and transmitter release (8–11).

DOA is an autosomal disease that affects the optic nerves, leading to reduced visual acuity and preadolescent blindness (12). The most common cause of DOA is mutations in *OPA1* that code for a dynamin-related guanosine triphosphatase (13, 14). OPA1 is required for lipid mixing and fusion of the mitochondrial inner membranes (15). In addition to the optic nerve, *OPA1* has been shown to be expressed in the retina in the photoreceptor IS (16). The role and impact of its loss of function in photoreceptors has not been well studied.

In most published transmission electron microscopy (TEM) images of mouse photoreceptors, the tissue is usually orientated longitudinally. When viewed like this, it is difficult to determine the fine positioning of mitochondria and how this relates to the energy and storage demands within the photoreceptor IS. In this study, we set out to examine the mitochondria arrangement in detail throughout the depth of entire photoreceptor ISs, as well as the cristae architecture using three-dimensional (3D) electron microscopy analyses. We provide a description of extensive contact sites between mitochondria and the PM in mammalian cells and have discovered a striking alignment between the mitochondria and their cristae in neighboring IS, suggesting a form of communication between cells. Our demonstration of disrupted mitochondria:PM contacts, and loss of specialized arrangement, in the heterozygous *Opa1* knockout (KO) mouse model supports the potential importance of mitochondrial alignment in normal retinal function.

## Results

### Mitochondria from Neighboring Photoreceptor ISs Are Aligned to Run Side by Side.

**Fig. 1.**
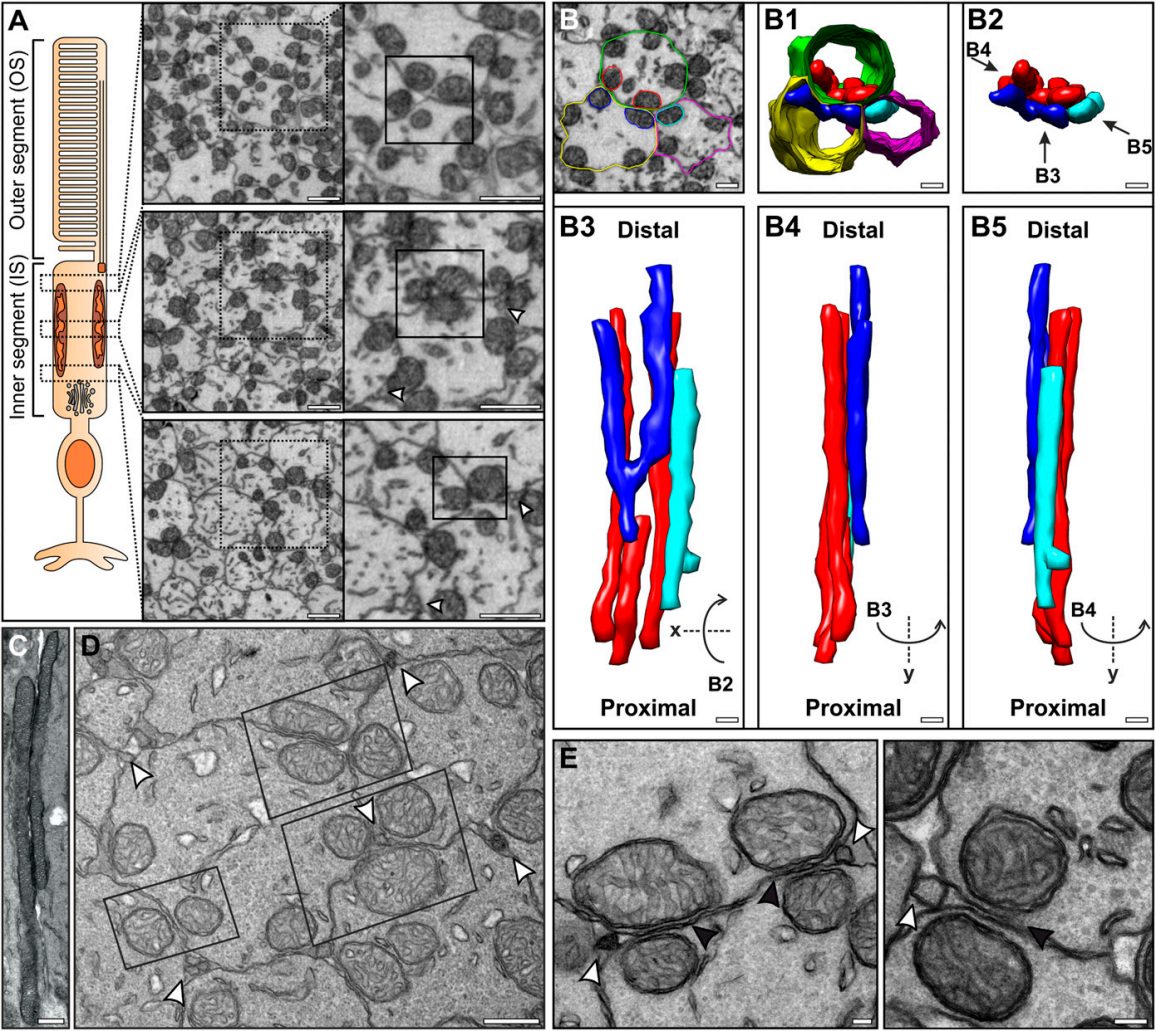
Mitochondria from neighboring photoreceptors align through the depth of the IS with projections visible nearby in the extracellular space. (*A*) SBFSEM images of mouse photoreceptors cross-sections where there is apparent alignment of mitochondria from neighboring photoreceptors that is most prominent in the midregion of the IS, as shown by the boxes with a solid outline. (*B*) Segmentation and modeling of select mitochondria and PMs from three ISs, showing the mitochondria running alongside each other. Top views of the model are shown in *B1* with and *B2* without the IS plasma membranes. The arrows in *B2 *indicate the point of view for the different side views of the model shown in *B3*–*B5*. (*C*) Longitudinal TEM section showing mitochondria from neighboring ISs running side by side. (*D*) TEM cross-section showing mitochondria arranged in pairs or triplets between neighboring ISs, highlighted by the boxed regions. Membrane projections were seen in between the ISs in close proximity to the mitochondria (white arrowheads). These are shown at a higher magnification in *E* by the white arrowheads, as well as alignment of neighboring IS mitochondria indicated by the black arrowheads. (Scale bars: 1 µm [*A*], 500 nm [*B*–*D*], and 100 nm [*E*].)

To study the 3D arrangement of mitochondria within photoreceptor ISs, postnatal day 20 (P20) wild-type mouse eyes were prepared for serial block face-scanning electron microscopy (SBFSEM). Single images from the SBFSEM data showed the mitochondria to be positioned in close proximity to the PM throughout the entire IS (Fig. 1*A*). The arrangement of mitochondria varied at different depths of the IS. Within most of the IS, mitochondria appeared to cluster adjacent to mitochondria in neighboring photoreceptors. Reduced clustering was observed at the proximal (Golgi region) tip of the IS (29.44% ± 8.87% SE) compared to the distal (close to the OS) tip (53.18% ± 3.69% SE) and the middle of the IS (62.21% ± 3.60% SE) (*SI Appendix*, Fig. S1). By modeling a portion of SBFSEM data, the mitochondria from neighboring photoreceptors can be seen to clearly run alongside each other through most of the depth of the IS (Fig. 1*B*, *SI Appendix*, Fig. S2, and Movies S1 and S2). Some mitochondria were seen to be fork-shaped and run alongside multiple mitochondria from neighboring cells (as shown by mitochondria modeled in blue in Fig. 1*B*). The mitochondrial arrangement determined from the SBFSEM data correlated with observations made from conventional TEM samples of 6-mo-old and P20 wild-type mice (Fig. 1 *C* and *D*). Mitochondria from neighboring photoreceptors were seen running side by side in longitudinal samples, as well as arranged in doublets or triplets in transversely orientated samples. The same mitochondrial arrangement was observed in rod and neighboring cone photoreceptors (*SI Appendix*, Fig. S3) that were identified by the higher density of less electron-dense mitochondria (17). In the extracellular space between photoreceptor ISs, often positioned between the mitochondria, small, circular membranous structures were observed within TEM images (white arrowheads in Fig. 1 *D* and *E*). When examined in the SBFSEM data, these were found to be projections that run up between the ISs (*SI Appendix*, Fig. S2).

### Mitochondria Are Tethered to the PM, and the Cristae between Mitochondria from Neighboring Cells Appear to be Aligned.

**Fig. 2.**
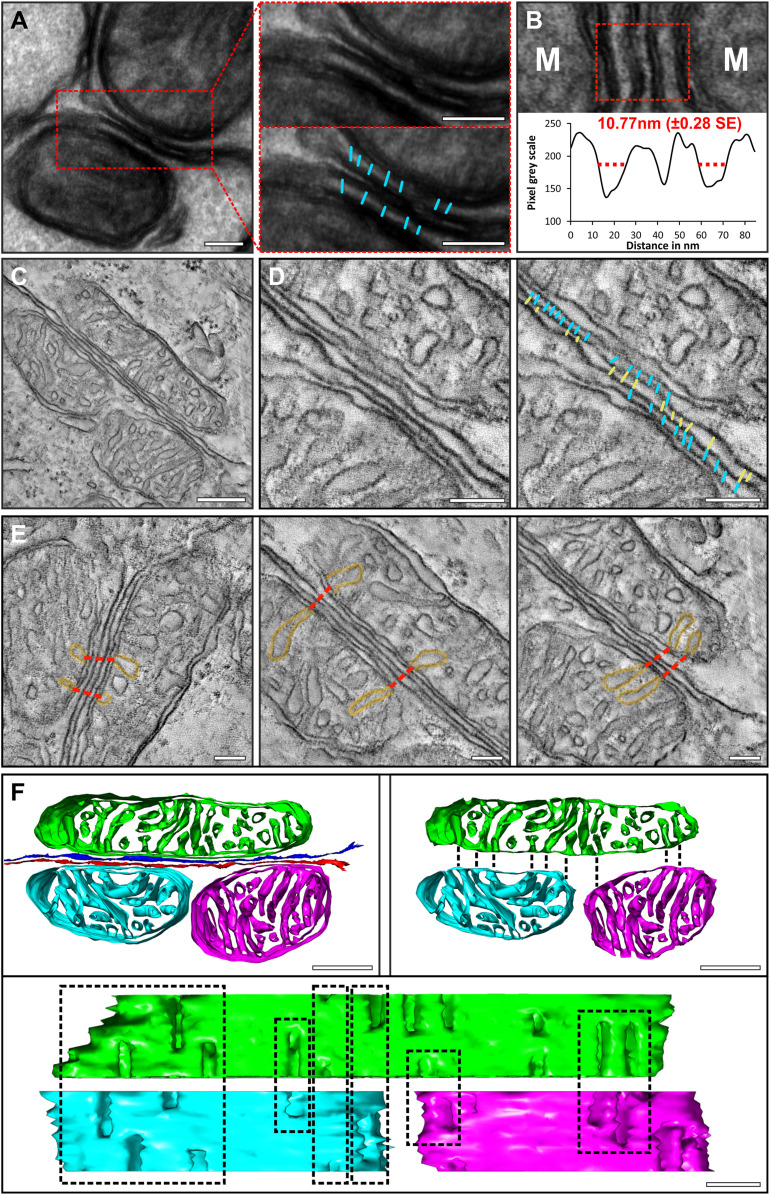
Visible tethers connect mitochondria to the IS PM, and there is alignment of the mitochondrial cristae between neighboring cells. (*A*) Tethers between the mitochondrial outer membrane and the PM are visible in a thick (200-nm) TEM section. The red box in *A*, *Left*, is shown at higher magnification in the two boxes in *A*, *Right*, with one box including an overlay of the tethers in cyan. (*B*) The distance between the mitochondrial outer membrane and PM is well conserved at 10.77 nm (±0.28 SE from 41 mitochondria). (*C*–*F*) Tomography reconstructions and resulting models of IS mitochondria. (*C* and *D*) A single slice from a tomogram reconstruction (*C*), and when focusing on the PM (*D*), tethers can be seen connecting the mitochondria to the PM (cyan) as well as structure detected between the two ISs (yellow). (*E*) Single slices from the tomograms showing alignment of cristae openings of mitochondria from neighboring photoreceptors. The dotted red lines indicated the path between aligned cristae openings. (*F*) A model of the mitochondria generated from a tomogram reconstruction. When removing the outer membrane from the model, there is visible alignment of the cristae openings as indicated by the boxes. (Scale bars: 100 nm [*A*], 250 nm [*C*], and 100 nm [*D*–*F*].)

By examining the IS mitochondria at high magnification within TEM images, electron-dense tethers were detected between the mitochondrial outer membrane and the PM (Fig. 2*A* and *SI Appendix*, Fig. S4). In addition, a high degree of consistency was found when measuring the distance between the two membranes (Fig. 2*B*), which was found to be 10.77 nm (±0.28). To examine the tethering and mitochondria structure in 3D at a higher resolution than is achievable by SBFSEM, tomograms were generated (Fig. 2*C*). Within the tomographic data, the tethers were resolved and could be seen bridging the outer mitochondrial membrane and PM (Fig. 2*D* and Movies S3 and S4). When examining the mitochondrial cristae, at different depths within the tomograms, the openings of the cristae appeared to be aligned (77.84% ± 0.98% SE aligned openings measured from five tomograms) between neighboring mitochondria (Fig. 2*E*). This was further confirmed by 3D modeling of the mitochondria membranes, indicating that many of the cristae openings are opposed to each other (black dotted line in Fig. 2*F* and Movies S3–S5).

### Neighboring Cell Mitochondrial Cristae Alignment Was Not Observed within the Retinal Pigment Epithelium.

**Fig. 3.**
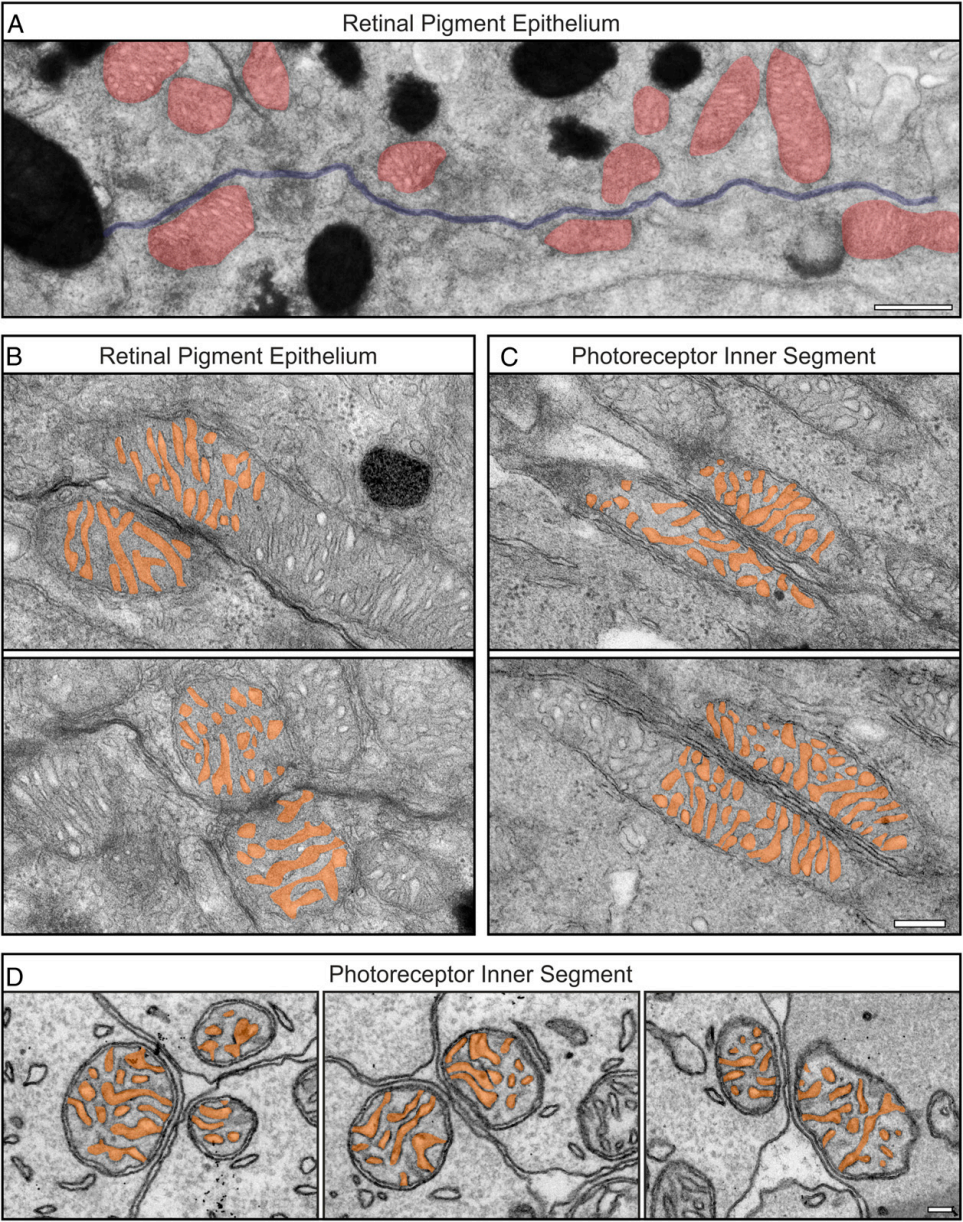
The mitochondrial alignment is not observed in the RPE when compared to photoreceptor IS. (*A*) At the lateral RPE border mitochondria (in red) are in contact with the PM (in blue). (*B*) TEM image of mitochondria from neighboring RPE cells including false-colored cristae show little alignment of the cristae openings. (*C* and *D*) The cristae opening alignment is seen in longitudinally orientated (*C*) and transverse (*D*) TEM sections of neighboring IS mitochondria. (Scale bars: 500 nm [*A*], 200 nm [*B* and *C*], and 100 nm [*D*].)

To investigate if the alignment of the cristae across mitochondria of neighboring cells is a general phenomenon in the retina, the retinal pigment epithelium (RPE) was examined. At the RPE lateral membrane, mitochondria are closely associated with the PM (Fig. 3*A*; distance between mitochondrial outer membrane and PM for 102 mitochondria measured as 10.45 ± 0.53 nm SE), similar to what we observed in the photoreceptor IS. Most of these were not found to be positioned adjacent to mitochondria of neighboring cells (42.01% ± 5.25% SE adjacent to neighboring mitochondria), and the ones that were had little or no cristae alignment (Fig. 3*B*). This was in contrast to the photoreceptor IS cristae that were found to be aligned in both longitudinally and transversely orientated tissue samples (Fig. 3 *C* and *D*).

### Müller Glial Processes Run between Photoreceptor ISs.

**Fig. 4.**
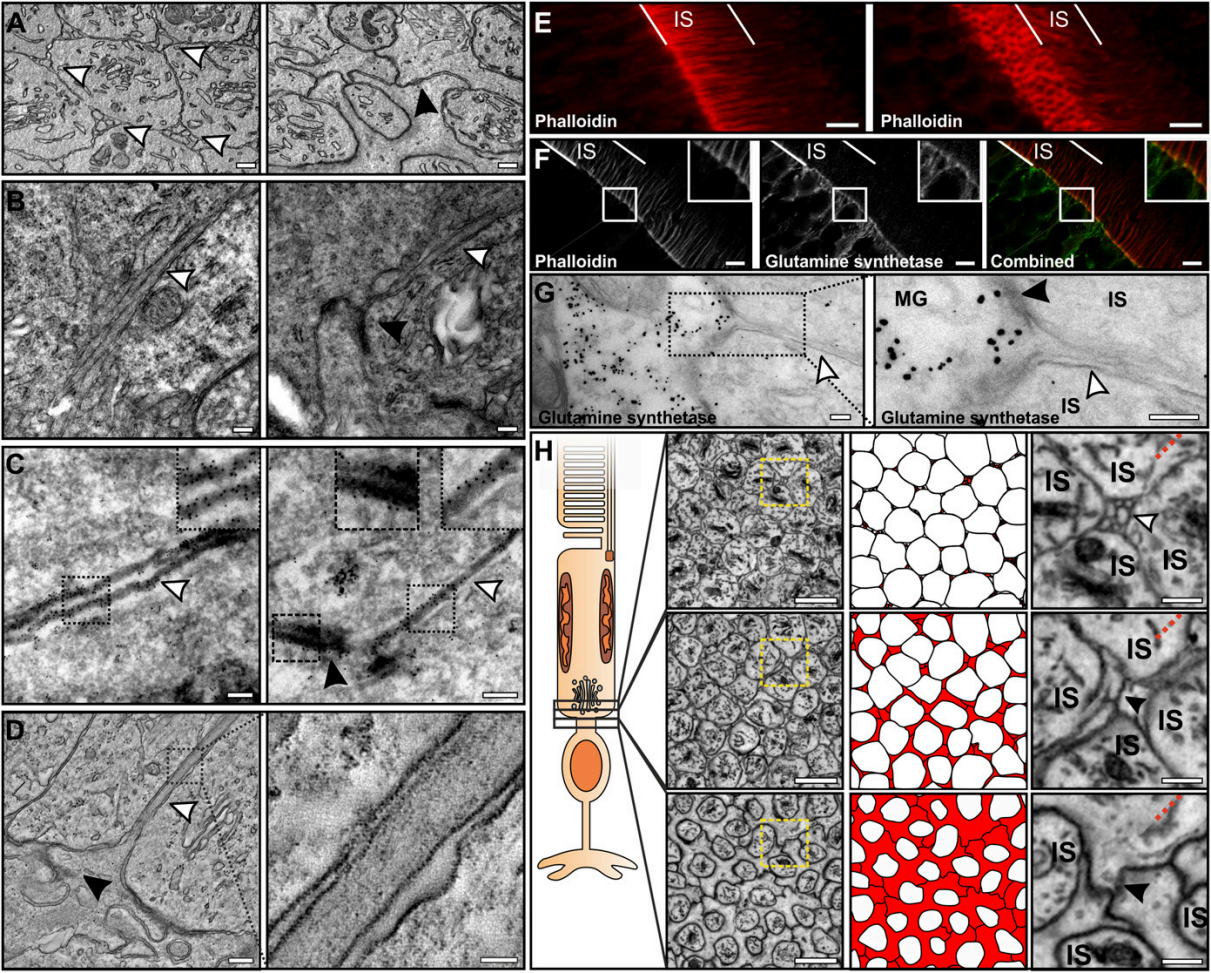
Projections that run between the ISs are actin-rich and are Müller glial cell-derived. (*A*) TEM images of cross-sections at the proximal region of the IS. In *A*, *Left*, projections run between the ISs (white arrowheads), and *A*, *Right* is closer to the photoreceptor cell body, where the projections are no longer visible, and junctions between the ISs are seen (black arrowhead). (*B*) Longitudinal images of the projections (indicated by white arrowheads) running between the ISs (black arrowhead shows the presence of junctions). (*C*) ImmunoEM labeling using an antibody against β-actin labeling indicates that it is enriched within the projections (white arrowheads) and at junctions (black arrowhead). Higher magnification images are shown in the corresponding boxed regions. (*D*) A slice from a tomographic reconstruction resolves the filamentous content of the projections (white arrowhead). The box in *D, Left* corresponds to the higher magnification image of the projection shown in *D, Right.* (*E*) Confocal stack of phalloidin-stained retina stains the projections, and when tilting the stack (*Right*), a “honeycomb” pattern represents the actin enrichment at the junctions (shown by black arrowheads within the TEM images). (*F*) Immunofluorescent antibody labeling of phalloidin and the Müller glial marker glutamine synthetase that have little localization. The larger white squares show higher magnification images of the selected regions (smaller white squares). (*G*) ImmunoEM labeling for glutamine synthetase shows the projections that lack labeling extend from the labeled Müller glial cells. A higher magnification image of the boxed region in *G, Left* is provided in *G, Right*. The white arrowheads indicate the position of a projection and black arrowhead shows the presence of a junction. (*H*) SBFSEM images show that the projections (colored in red in *Middle*) originate from the Müller glial cells surrounding the photoreceptors in the ONL. The yellow box in *H, Left,* is shown at higher magnification in *H, Right* and the white arrowhead indicates the position of the projections and the black arrowheads show the presence of junctions between cells. The red dotted lines in *H*, *Right* indicate the position of image distortion resulting from slight rotation of the SBFSEM image stack in 3D to reduce obliqueness of the inner segment. (Scale bars: 250 nm [*A*–*D*], 5 µm [*E* and *F*], 200 nm [*G*], 2 µm [*H*, *Left*], and 500 nm [*H*, *Right*].)

The small circular membranes observed in transversely orientated mouse retina (Fig. 4*A*) were seen as tubular projections running between photoreceptor ISs when viewed in longitudinally orientated samples (Fig. 4*B*). Images from SBFSEM data showed that these originated at the border between the outer nuclear layer (ONL) and the IS layer and run up to approximately half the length of the IS (*SI Appendix*, Figs. S2 and S5*A*). When performing immuno-electron microscopy (immunoEM) labeling for actin (anti–β-actin in Fig. 4*C* and phalloidin staining in *SI Appendix*, Fig. S5*B*), the staining was enriched within these projections as well as at membrane junctions at the proximal IS (white and black arrowheads, respectively, in Fig. 4*C*). In contrast to the actin enrichment in the projections between the ISs, no cortical actin staining was detected at the lateral borders. A tomogram resolved the filamentous content running through the projection (Fig. 4*D*), which was likely to be actin filaments. Labeling F-actin with phalloidin highlighted the actin-enriched projections within the IS layer, and when tilting the 3D confocal data, a “honeycomb”-like pattern was observed, reflecting the membrane junctions at the proximal IS (Fig. 4*E* and *SI Appendix*, Fig. S5*C* and black arrowheads in Fig. 4 *A*–*D*). To determine the origin of the projections, an antibody against glutamine synthetase was used, as it is a well-known Müller glial cell marker. By immunofluorescence (IF), there was no observed colocalization other than an overlapping region of enriched staining at the base of the projections (phalloidin staining) and within the glutamine synthetase channel (Fig. 4*F*, small panels). To investigate this further, immunoEM labeling against glutamine synthetase was performed on retinal sections. This clearly showed that the projections emanated from the labeled Müller glial cells (Fig. 4*G*; and further staining in *SI Appendix*, Fig. S5*D*). In agreement with the IF staining, the glutamine synthetase was absent from the projections in the immunoEM-labeled sections and only present within the cell body. By highlighting the projections within the SBFSEM data, they could be traced to the cells that surrounded the photoreceptor rather than the photoreceptors themselves (Fig. 4*H*), providing further evidence they are Müller glial cell-derived.

### Mitochondria Arrange against the PM and Aligned between Neighboring ISs by P20.

**Fig. 5.**
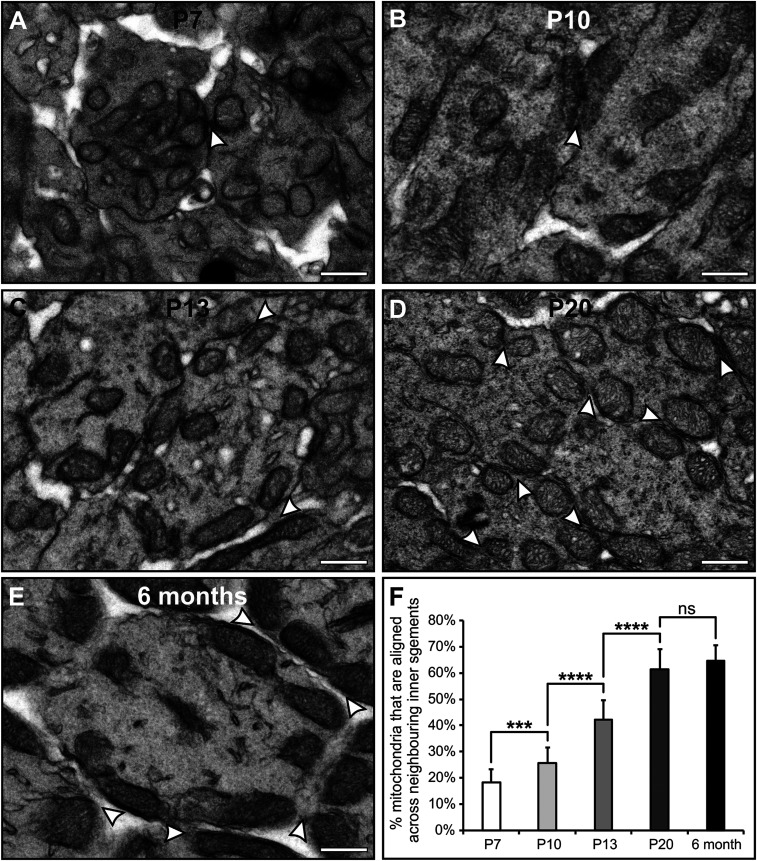
The mitochondria of neighboring ISs are not aligned until P20. (*A*–*E*) TEM images of ISs from mice of ages P7 (*A*), P10 (*B*), P13 (*C*), P20 (*D*), and 6 mo (*E*). (*F*) There is increasing alignment of mitochondria between neighboring ISs up to P20 (average = 15 regions with ≥158 mitochondria from three eyes). White arrowheads indicate alignment of neighboring IS mitochondria. (Scale bars: 500 nm). ***P < 0.001; ****P < 0.0001; ns, not significant (determined by Student's *t*-test).

To determine the stage at which neighboring photoreceptor IS mitochondria align, we examined mouse ISs (randomly assessed, as shown in *SI Appendix*, Fig. S6) at different ages. At P7, the immature photoreceptors (identified in the central retina by the presence of immature OSs; example labeled in *SI Appendix*, Fig. S7) have a short IS consisting of scattered mitochondria, some in contact with the PM, as well as others positioned away from the PM (Fig. 5*A*). At P7, we observed the presence of Müller glial processes (*SI Appendix*, Fig. S7), but there was little alignment between mitochondria of neighboring ISs (18.17% ± 5.15% SE). By P10, a greater number of mitochondria were observed positioned against the PM, with some still positioned centrally within the IS (Fig. 5*B*). At P13, greater alignment between neighboring IS mitochondria was observed (Fig. 5*C*), but it was not until P20 that the alignment reflected that of mature retina at 6 mo (Fig. 5 *D*–*F*). To determine if alignment of mitochondria from neighboring ISs could be due to chance, assessment of randomly selected ISs and mitochondria was undertaken, and a theoretical probability model was generated (*SI Appendix*, *Supplementary Methods* and Figs. S8–S11). As ISs were found to contain between zero and seven mitochondria, this provided eight IS types, and the resulting model was set up to use idealized values, as shown in the table in *SI Appendix*, Fig. S11 *A* and *B*. The model was validated against hexagonal array simulations and was shown to generate similar values (*SI Appendix*, Figs. S9 and S10). Testing the model with increasing mitochondrial size provided evidence that the arrangement of the mitochondria is highly unlikely to be due to random placement, as 65.2% of mitochondria were found to be aligned to a neighboring IS mitochondria within the mid-IS region, as measured from TEM data, whereas 27.3% (when setting the model mitochondrial diameter to the equivalent of the real IS average mitochondrial long-axis diameter) was calculated to be due to random placement from the probability model (*SI Appendix*, Figs. S8 and S11).

### Heterozygous KO of *Opa1 *Alters Mitochondrial Positing, but Does Not Affect Cristae Alignment.

**Fig. 6.**
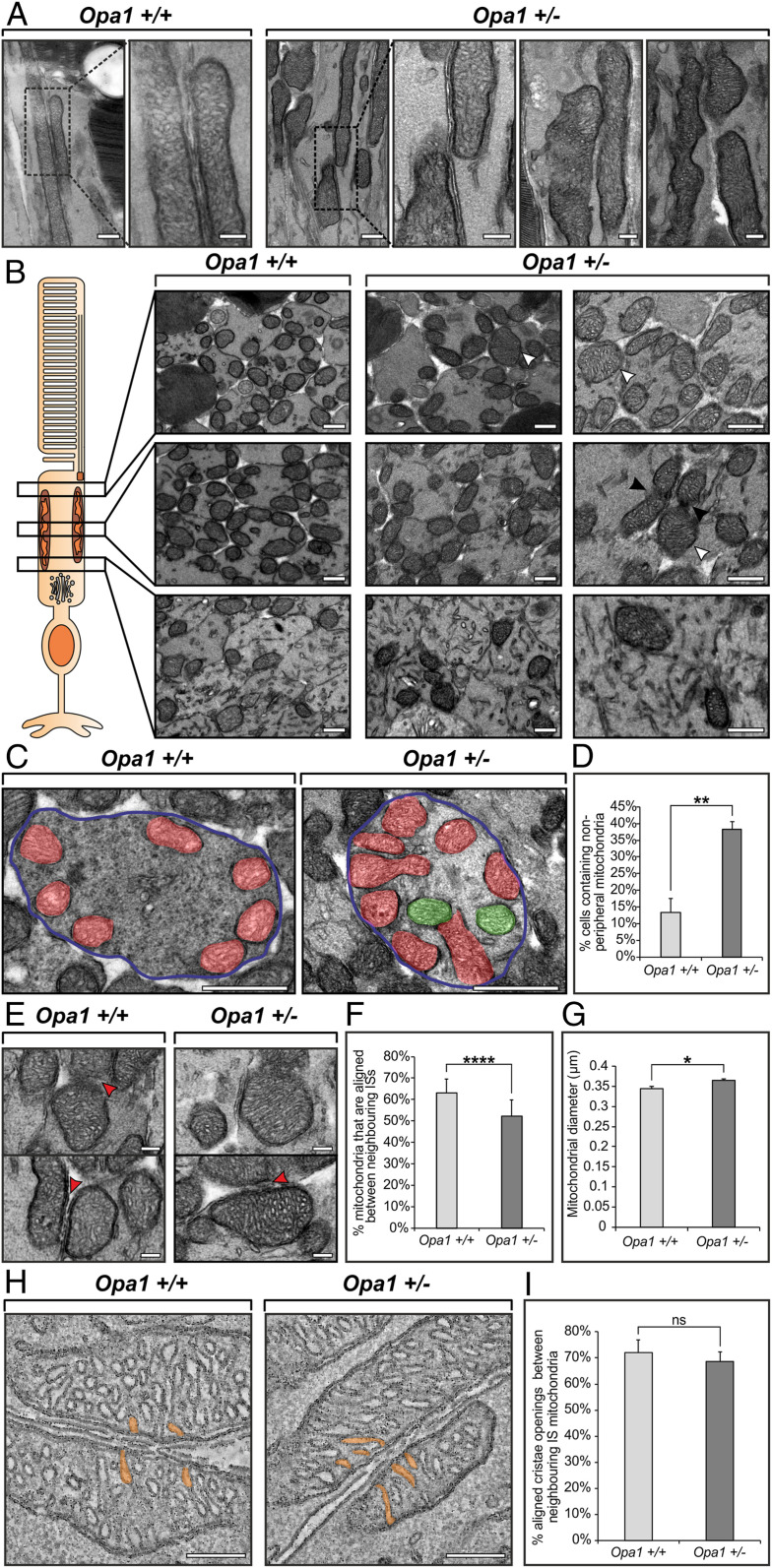
Heterozygous KO of *Opa1* leads to changes in the mitochondria morphology and positioning, but not the alignment of cristae openings. (*A*) TEM images of longitudinally orientated retina from *Opa1*^+/+^ and *Opa1*^+/−^ mice. The boxed areas are shown at higher magnification in the panels on the right. (*B*) Cross-sectionally-orientated retina examined at different regions of the IS indicate enlarged mitochondria (white arrowheads) and evidence of a mitochondrial fusion defect (black arrowheads). (*C*) IS cross-sections in which mitochondria against the PM are false colored in red, and those away from the PM are in green. (*D*) An increase in the number of ISs that have mitochondria positioned away from the PM in the *Opa1*^*+*/−^ (average = three eyes). (*E*) Images showing larger mitochondria in the *Opa1^+^*^/−^ mice ISs, but no obvious morphological defects of the cristae. The red arrowheads indicate where there are mitochondria aligned between neighbouring ISs. (*F*) A reduced number of mitochondria are aligned between neighboring ISs (average = 15 regions with ≥127 mitochondria from three eyes). (*G*) Measurements of mitochondria show an increase in the average diameter in the *Opa1*^+/−^ mice (average = 300 mitochondria from three eyes). (*H* and *I*) Tomographic slices show the presence of cristae opening alignment in the both the *Opa1*^+/+^ and *Opa1*^*+*/−^ mice, and measurement show no significant difference between the two mouse models (average = three tomograms). (*E*–*G*) Data are mean ± SE. *P < 0.05; **P < 0.01; ****P < 0.0001; ns, not significant (determined by Student's *t* test). (Scale bars: 200 nm [*A*], 500 nm [*B*], 1 µm [*C*], and 200 nm [*E* and *H*]).

As OPA1 is known to play a role in mitochondrial fusion and cristae morphology (18), and loss of function is associated with specific deficits in visual electrophysiology (19), we examined the eyes of heterozygous KO mice by TEM. From longitudinally orientated retinal samples, the ISs of *Opa1*^*+/−*^ mice presented misshaped mitochondria that had reduced alignment to neighboring IS mitochondria when compared to *Opa1*^*+/+*^ (Fig. 6*A*). Retinal cross-sections at different depths of the ISs showed some mitochondria in the *Opa1*^*+/−*^ mice to be larger or abnormally shaped compared to those from *Opa1*^*+/+*^ mice (Fig. 6*B*). The positioning of mitochondria within the ISs and the mitochondrial diameter were quantified from photoreceptor cross-sections (Fig. 6 *C*–*G*). The percentage of ISs containing mitochondria positioned away from the PM was higher in the *Opa1*^*+/−*^ (38.32% ± 2.31% SE) compared to the *Opa1*^*+/+*^ (13.25% ± 4.22% SE) mouse eyes (Fig. 6 *C* and *D*). Measurements of the shortest mitochondrial diameter as well as the alignment between neighboring IS mitochondria indicated that the *Opa1*^*+/−*^ mouse IS had larger mitochondria and reduced alignment compared to the *Opa1*^*+/+*^ mice (Fig. 6 *E*–*G* and *SI Appendix*, Fig. S12*A*). Tomographic reconstructions were generated to examine the mitochondrial cristae (Fig. 6*H*). Within the tomographic slices, cristae openings were found to be aligned in mitochondria that were positioned against the PM in both the *Opa1*^*+/−*^ and *Opa1*^*+/+*^ ISs (Fig. 6 *H* and *I* and *SI Appendix*, Fig. S12*B*). Large mitochondria positioned away from the PM in the *Opa1*^*+/−*^ ISs did not appear to have unusual or disordered cristae (*SI Appendix*, Fig. S12*B*).

## Discussion

The high energy demands and importance of mitochondria for calcium storage in photoreceptors are well established (1, 11). Yet, the arrangement of mitochondria within photoreceptor ISs has not been well studied. This has largely been hampered by the lack of techniques to image through entire ISs at the resolution that is achievable by TEM. The development of SBFSEM methodology allows serial imaging through large tissue volumes and is ideal for examining photoreceptor mitochondria. This combined with a range of electron microscopy techniques has allowed us to make a number of important findings in regard to the highly specialized mitochondrial arrangement in the IS, shedding light on how this may help regulate energy and metabolite homeostasis across the mouse photoreceptor cell layer.

When examining through the depth of mouse photoreceptor ISs, mitochondria remained in contact with the PM and were seen to cluster together in pairs or triplets. The clustered mitochondria were found to be aligned to each other, running alongside most of the length of the IS, resulting in large mitochondrial surface areas facing each other. By developing a probability model, we show that this alignment is unlikely to be a result of random mitochondrial placement. The distance between the mitochondria outer membrane and the PM was highly consistent, and we observed this to be maintained by tethering between the membranes. Mitochondria–PM contact sites have been identified in yeast (20), but have never before been described in mammalian cells. At mammalian brain synapses (21), filamentous connections, described as cytoskeletal anchors, have been reported to connect mitochondria with adherens junctions, but may not be linked directly to the PM. Furthermore, these synaptic filamentous links were >30 nm in length, in contrast to the ∼11-nm tethers that we have shown here, which are consistent with the length of previously characterized tethers at membrane contact sites (22–24). As there is no known mammalian homolog of the yeast mitochondria–PM tethering components Num1 and Mdm36, as well as a lack of a reliable in vitro photoreceptor model with fully differentiated OSs, we were unable to determine the constituents of the IS PM tethers (20, 21). The close association of the mitochondria to the PM and the alignment to mitochondria from neighboring photoreceptors implies that there is communication and/or sharing of resources. Photoreceptors are highly sensitive to hypoxia and nutrient deprivation, but the pathway by which they can maintain the metabolite levels to meet their high energy demands is not fully understood (25). Furthermore, studies examining metabolic flux in the retina indicate the need for energy homeostasis across the photoreceptor cell layer and with the RPE to maintain retinal health and visual acuity (5, 26–28). Therefore, the mitochondria arrangement described in this study may be an important evolutionary development for sharing particular metabolites across the photoreceptor cell layer. It is possible the position of the mitochondria at the PM assists in directing light, as there is evidence that nocturnal mammals have a nuclear architecture consisting of heterochromatin localized in the center that directs light up through the IS to the OS (29). Further work is required to test these hypotheses, using techniques to measure levels of metabolites, as well as energy metabolism, from photoreceptor cells in different mammalian models that have a clear mitochondrial disarrangement phenotype or disrupted mitochondrial tethering to the PM.

Tomographic reconstructions of IS mitochondria were used to resolve the fine cristae architecture. Single slices from the data as well as segmentation models showed a high degree of alignment of cristae openings between mitochondria of neighboring cells. To determine if these were coincidental or exist in other cell types, RPE mitochondria were inspected. Mitochondria at the lateral RPE cell border were found to be in contact with the PM, similar to what we observed in ISs, but most mitochondria from neighboring cells were not found to be positioned side by side. Due to the conserved distance, we predict that there are tethers to the RPE lateral cell border, but in this study, we were unable to clearly detect if they were present. When examining the cristae from the few neighboring RPE cell mitochondria that were observed, the cristae showed little, if any, alignment when compared to what was observed in the IS. The alignment of the cristae openings further supports the notion of a mechanism for communication and/or exchange of resources across PMs to mitochondria of neighboring cells. Mitochondria within mouse cardiomyocytes and human skeletal muscle have been shown to form dense intermitochondrial junctions that have alignment/coordination of the cristae (30, 31). Here, we were able to show cristae alignment between mitochondria from neighboring cells. It is possible in photoreceptors that the mitochondria are polarized so that the cristae face the PM and the cristae openings are separated evenly, which gives the appearance of alignment. The tomography slices and model indicate that this is unlikely, however, as the openings do not appear to be evenly distributed and have a similar pattern of openings when compared to the opposing mitochondrial inner membranes. Furthermore, the shape of the cristae in some of the TEM images demonstrate them curving into position for alignment to a neighboring mitochondrion. For the cristae to align across cells would require a coordinated complex at contact sites where the cristae openings are positioned. It is known at the cristae opening that there is coordination and contacts between the inner and outer mitochondria membrane, leading to the positioning of particular channels (32–34). At these sites on the outer mitochondrial membrane, there would likely need to be further connections bridging to the PM, where there is another process that coordinates corresponding tethers within the neighboring cells. For this to occur, it is expected there would be either 1) further tethers between cells similar to those we observed in tomography slices (Fig. 2*C*), 2) stimulation by release of metabolites/protein through PM channels, or 3) PM lipid-enriched domains that are coordinated by the mitochondria to be positioned to line up with cristae openings.

In the extracellular spaces between photoreceptors, often positioned close to the IS mitochondria, tube-like projections were observed. In previous studies, these have been proposed to be Müller glial processes, but there has been a lack of definitive evidence at the resolution of TEM to prove this to be the case (35, 36). To better characterize as well as determine the origin of these projections, we used a combination of immunolabeling and 3D electron microscopy techniques. We found that they were actin-enriched, when staining for actin by immunoEM, and, while doing so, we did not detect cortical actin at the lateral border of the photoreceptor IS. The latter finding correlates well with our mitochondria observations within the IS, as actin filaments would likely hinder the positioning and tethering of mitochondria to the PM. By staining for glutamine synthetase, a well-known Müller glial cell marker, it was found to be absent from the projections, but labeled the cells that the projections emanated from. In addition, imaging through the depth of the retina close to the IS–ONL junction by SBFSEM demonstrated that the projections do not originate from photoreceptors and unequivocally showed that they are Müller glial cell-derived. It has been proposed in retina that there is an metabolic ecosystem, and, in addition to the well-established metabolic transport between the RPE and photoreceptors (37–39), Müller glial cells are involved in shuttling of lactate as well as other metabolites (5). Due to the positioning of the Müller glial processes close to the IS mitochondria, they may exist to assist in transport of resources toward or away from the IS mitochondria. The actin filaments within the processes likely exist to support and stabilize the structure and other systems are involved in the transport of resources.

To determine the developmental stage at which the mitochondria within the IS contact the PM and align to neighboring cell mitochondria, mouse eyes with developing photoreceptors were examined. At the youngest timepoint, P7, the mitochondria appeared dispersed and showed progressive rearrangement at successive timepoints until P20, when the mitochondria were positioned in a similar arrangement to adult 6-mo-old retina. The outer limiting membrane layer that forms close to the proximal IS has formed by P7, as Müller glial processes were observed, indicating that their presence is independent of the mitochondrial rearrangement.

To determine the effect of reducing a known regulator of mitochondrial structure, OPA1, heterozygous *Opa1* KO mouse photoreceptors were examined. In the IS, there was evidence of a fusion defect in some mitochondria, but, more strikingly, the mitochondria were larger, and a greater proportion were positioned away from the PM with reduced alignment to neighboring IS mitochondria in the *Opa1*^*+/−*^ compared to the *Opa1*^*+/+*^ mice. The cristae morphology and openings appeared unaffected, and those mitochondria that were in contact with the PM were aligned with neighboring cell mitochondria in the *Opa1*^*+/−*^ mice. OPA1 is most highly expressed in the retina, and, in heterozygous KO, the expression levels may have been adequate to provide normal cristae morphology, in combination with other factors that are regulating the cristae opening alignment (40). The reduced proportion of mitochondria in contact with the PM makes the *Opa1*^*+/−*^ a good model for future studies to determine the pathway that leads to the specialized IS mitochondrial arrangement.

This study sheds light on how the position and morphology of mitochondria have likely evolved to fulfill the energy and storage demands across the photoreceptor cell layer and provides evidence for a form of mitochondria-mediated inter cellular communication. Identification of the mitochondria:PM tethers and the factors regulating the cristae alignment will allow the establishment of the nature of this communication and its importance in other tissues.

## Materials and Methods

Mouse eyes were processed for SBFSEM, TEM, tomography, immunoEM, and IF as described in detail in *SI Appendix*. The eyes used were from mice that had been killed by cervical dislocation in accordance with Home Office (United Kingdom) guidance rules under project licenses 70/8101 and 30/3268. This was undertaken adhering to the Association for Research in Vision and Ophthalmology (ARVO) Statement for the Use of Animals in Ophthalmic and Vision Research (https://www.arvo.org). Heterozygous *Opa1 *KO mice were generated as described (41).

### Data Availability.

Tomographic data are available at the Electron Microscopy Data Bank, www.emdataresource.org/ (accession no. EMD-10904 [IS mitochondria], EMD-11126 [IS mitochondria], EMD-11125 [Müller glial projection], EMD-11148 [*Opa1*^+/+^ IS mitochondria], EMD-11147 [*Opa1*^+/−^ IS mitochondria]).

## Supplementary Material

Supplementary File

Supplementary File

Supplementary File

Supplementary File

Supplementary File

Supplementary File
