# Supplementary material for: Symmetric arrangement of mitochondria:plasma membrane contacts between adjacent photoreceptor cells regulated by Opa1
Source: Proc Natl Acad Sci U S A. 2020 Jun 22;117(27):15684–93. doi: 10.1073/pnas.2000304117 (PMC7355040; doi:10.1073/pnas.2000304117)
Supplement: Supplementary File [file pnas.2000304117.sapp.pdf]

PNAS
www.pnas.org

Supplementary Information for

Symmetric arrangement of mitochondria:plasma membrane contacts between adjacent photoreceptor cells regulated by Opa1

Ingrid P. Meschede, Nicholas C. Ovenden, Miguel C. Seabra, Clare E. Futter, Marcela Votruba, Michael E. Cheetham, Thomas Burgoyne

Corresponding author: Thomas Burgoyne
Email: t.burgoyne@ucl.ac.uk

This PDF file includes:

Supplementary text
Figures S1 to S12
Legends for Movies S1 to S5
SI References

Other supplementary materials for this manuscript include the following:

Movies S1 to S5

www.pnas.org/cgi/doi/10.1073/pnas.2000304117

Supplementary Information Text

Supplementary Methods

Transmission electron microscopy and tomography. Mouse eyes were fixed and embedded as described (1). ~100nm thick sections were cut and stained using lead citrate before acquiring images on a JEOL 1400+ TEM equipped with a Gatan Orius SC1000B charge-coupled device camera. For tomography 10-nm gold particle solution (fiducial marker) was used to stain the sections before tilting the stage from ±60° in 1.5° increments using the SerialEM software (University of Colorado Boulder). The images were processed and tomograms generated using the IMOD tomography package (2).

3view serial block face scanning electron microscopy. Eyes were fixed in 3% (vol/vol) glutaraldehyde and 1% (wt/vol) paraformaldehyde in 0.08 M sodium cacodylate buffer, pH 7.4 for 2 hrs at room temperature before incubating in the following solutions; 1% aqueous osmium tetroxide and 1.5% potassium ferrocyanide at 4°C for 1 hours, 1% aqueous thiocarbohydrazide at room temperature for 20 mins and 2% aqueous osmium tetroxide at room temperature for 30 minutes. They were sequentially en bloc stained 1% (wt/vol) aqueous uranyl acetate at 4°C overnight followed by Walton's lead aspartate 30 mins at 60°C. The samples were dehydrated in an ethanol series followed by propylene oxide and infiltration in a mixture of propylene oxide and Durcupan ACM resin (1:1), before embedding in Durcupan ACM resin at 60° overnight. Blocks cut from the embedded specimens were mounted onto aluminum pins and coated with gold palladium. Using a Gatan 3View system (Gatan Inc, Abingdon, UK) and a Zeiss Sigma VP field emission scanning electron microscope (Zeiss, Cambridge, UK), images were acquired in between the sequential cutting away of 100nm thick section of the sample. The images were re-aligned using the StackReg plugin (EPFL) in ImageJ (NIH) and the images were modelled using the IMOD tomography package (2).

Cryo-immuno-electron microscopy. Mouse eyes were fixed in 4% (wt/vol) paraformaldehyde and 0.1% glutaraldehyde in 0.1 M phosphate buffer at pH 7.4 for 2hrs. The cornea and were lens removed before cutting the eye cup into small blocks and embedding them in 12% (wt/vol) gelatin, followed by infusion with 2.3 M sucrose solution at 4 °C overnight. 80nm sections were cut at −120 °C and collected in 1:1 mixture of 2.3 M sucrose/2% (wt/vol) methylcellulose, and labeling was performed as described previously (3). Labelling of actin was performed using an anti-β-actin antibody (Sigma) or phalloidin bound to biotin (Molecular Probes) and anti-biotin (Rockland), followed by protein-A-gold (CMC, University Medical Center Utrecht).

Immunofluorescence. The eyes were fixed in 4% (wt/vol) paraformaldehyde in PBS for 2 hrs before infusing with 30% (wt/vol) sucrose at 4°C before embedding in OCT compound and freezing using a bath of acetone cooled to -78°C. 5–20 um sections of the frozen samples were cut at -20°C using a cryostat. The sections were permeabilised using 0.1% (vol/vol) saponin in PBS for 30 mins at room temperature being antibody labelling in blocking solution consisting 0.01% (vol/vol) saponin in 1% (wt/vol) BSA in PBS for 2 hrs at room temperature. The sections were incubated in the following primary antibodies; glutamine synthetase (Novus Biologicals) and RETP1 (Abcam). Secondary antibodies bound to an Alexa Fluor dye (ThermoFisher Scientific) were applied to the sections for 1 hr at room temperature before mounting mounted with ProLong Gold antifade reagent (Life Technologies) that contained DAPI to counterstain the nuclei. Images were taken on a Leica SP8 confocal microscope.

Immuno-electron microscopy of cryostat sections. Mouse eyes were prepared following the methods described here (4). In brief cryostat sections were permeabilised using 0.05% (vol/vol) triton in PBS for 30 mins at room temperature and using a blocking solution containing 1%

(wt/vol) BSA and 0.1% acetylated BSA in PBS, antibody labelling was performed using anti-glutamine synthetase (Novus Biologicals) for 2 hrs at room temperature. A secondary anti-rabbit bound to nano-gold (Nanoprobes) was applied in blocking solution for 2 hrs at room temperature before fixing the sections in 2% (vol/vol) glutaraldehyde, 2% (wt/vol) paraformaldehyde in 0.15 M sodium cacodylate buffer, pH 7.4 for 1 hr at room temperature. A gold enhance solution prepared in accordance with manufacturers specifications (Nanoprobes) and applied to the sections at 4°C for 10 mins. The sections were incubated in 1% (wt/vol) osmium tetroxide/1.5% (wt/vol) potassium ferrocyanide in distilled water for 1 hr at 4°C, before dehydrating in an ethanol series and embedding in epon at 60°C overnight. ~100nm thick sections were cut and images acquired on a JEOL 1400+ TEM with a Gatan Orius SC1000B charge-coupled device camera.

Image analysis. Electron microscopy and confocal images were analysed and measurements were made using ImageJ (NIH). To randomly assess inner segments and mitochondria grid was overlaid onto TEM images in ImageJ (see Fig S1, S6 and S8) and a random number generator in Microsoft Excel was used to pick random squares from the grid. Mitochondria alignment was classed as mitochondria positioned against the PM that overlapped with mitochondria similar position against the PM in a neighbouring IS. To quantify the number of cristae opening that were alignment between mitochondria, tomographic slices were examined using IMOD. The number of cristae opening facing the PM that either had or did not have a corresponding cristae opening in the alignment mitochondria in the neighbouring IS was determined.

Theoretical mitochondrial positioning model. Is it possible that the pattern of mitochondrial alignment observed arose by chance? To answer this, we constructed a theoretical model of random mitochondrial placement to determine whether the prevalence of mitochondrial alignment observed in the data could be a consequence of a random process of placement. The theoretical model is an idealised model of mitochondrial placement within each photoreceptor IS designed to replicate the experimental data. The model is based on the following assumptions:
There are N+1 IS types identified by the number of mitochondria contained within. Thus type k IS contains k mitochondria for k=0,1,...,N. IS type k also has a given prevalence P_k based on the experimental data (see table in Fig S11 A) and the types are distributed uniformly across the retina. Based on the data, each IS type k has a given perimeter length L_k and a given number of nearest neighbours Ω_k, which is also equal to the number of junction points where three neighbouring IS meet.
The size of all mitochondria is assumed to be constant and it is assumed that all mitochondria reside on the boundary of the IS. Inside each IS there is a fixed finite number of locations around the boundary of the IS that the mitochondria can occupy. The size of these locations reflects the approximate size of the mitochondria that potentially resides within. Hence, the number of locations thus approximates the ratio of the IS perimeter to the mitochondrial diameter. Within each IS type one location is situated at each junction (where three IS meet) and the other locations are situated along the IS edges.
Each of the k mitochondria within a given IS of type k are placed randomly in turn into one of the unoccupied boundary locations (Fig S9). Thus, the probability of finding x mitochondria (where x <= k) within a sample of m boundary locations from a population size given by the total number of locations L_k (where m<= L_k) is given by a Hypergeometric probability distribution function defined by Hy(x,L_k,k,m) (5), where

$$Hy(x, L_i, i, m) = \frac{\binom{i}{x}\binom{L_i - i}{m - x}}{\binom{L_i}{m}}$$

We take a random junction and then a random edge and assume that the IS types that form the junction/edge are randomly assigned independently of each other based on their prevalence. The length of the random edge (not inclusive of the junction locations) is given by the smallest IS, assuming its junction points are equally distributed around the perimeter. We then calculate the probabilities of mitochondrial alignment occurring at that junction or on that edge based on the Hypergeometric PDF governing random mitochondrial placement within each IS.
Based on these assumptions the probability of three randomly placed mitochondria aligning at a junction point given that the three neighbouring IS are of type i, j, k is

$$P(3 \text{ at a junction}|i,j,k) = Hy(1, L_i, i, 1)Hy(1, L_i, j, 1)Hy(1, L_i, k, 1)$$

On the other hand, the probability of two randomly placed mitochondria aligning at the same junction point is

$$P(2 \text{ at a junction}|i,j,k) = \sum_{s=1}^{3} Hy(1-\delta_{1s}, L_i, i, 1)\, Hy(1-\delta_{2s}, L_j, j, 1)Hy(1-\delta_{3s}, L_k, k, 1)$$

We can also determine the likelihood of s aligned mitochondria on a given edge between two random IS types i and j, where without loss of generality we define i to be the smallest, i.e. i<=j:

$$P(s \text{ aligned pairs on an edge}|i,j) = \sum_{k=s}^{i} Hy(k, L_i, i, E_i)Hy(s, L_j, j, k)$$

where

$$E_i = \frac{(L_i - \Omega_i)}{\Omega_i}$$

is the number of locations on the neighbouring edge based on smallest IS.
Finally, the conditional probability of zero aligned mitochondria occurring on the given edge is

$$P(\text{zero aligned pairs on an edge}|i,j) = Hy(0, L_i, i, E_i) + \sum_{k=1}^{i} Hy(k, L_i, i, E_i)Hy(0, L_j, j, k)$$

To obtain the overall probability of one of these occurring on a randomly selected edge or junction can thus be determined by summing the conditional probabilities multiplied by prevalences of each IS type being present. Hence,

$$P(3 \text{ occurring at a junction}) = \sum_{i=0}^{N}\sum_{j=0}^{N}\sum_{k=0}^{N} P(3 \text{ occurring at a junction}|i,j,k)P_iP_jP_k$$

and, similarly for instance,

$$P(\text{zero aligned pairs on an edge}) = \sum_{i=0}^{N}\sum_{j=0}^{N} P(\text{zero aligned pairs on an edge}|i,j,)P_iP_j$$

To benchmark this theoretical model we tested it for a single IS type, with six neighbouring IS and compared this to a MATLAB (MathWorks) code which places k circles (representing mitochondria) each at random into an empty one of n locations around the boundary of each hexagon in a hexagonal tiling. The results are shown in Fig S10 for different mitochondrial sizes (fewer locations) and different number of mitochondria per hexagon. Aligned IS at junctions and on edges are highlighted and the frequency of occurrence shows very good agreement with the probability model.

To calculate the expected % of aligned mitochondria in the theoretical models, the various probabilities of one or more aligned pairs occurring on an edge and of pairs/triplets occurring at junctions were multiplied by the number of internal edges or junctions in a tiling of M tiles respectively, and the number of aligned mitochondria in each instance (e.g. 2 for a pair, 3 for a triplet etc.). These values were then added together to yield the total number of mitochondria aligned and this total was then divided by the total number of mitochondria within the tiled space (kM) to obtain a percentage. For the results in Fig S11 C a further approximation was used where M was assumed to be large.

For a more accurate comparison with the data we developed an idealised set of 8 IS types based on the available experimental data as shown in see table in Fig S11 A & B. This led to the results shown in Fig S11 C using different numbers of perimeter locations to demonstrate that, even with enlarging mitochondrial size, there is less mitochondrial alignment in the models than compared to real ISs.

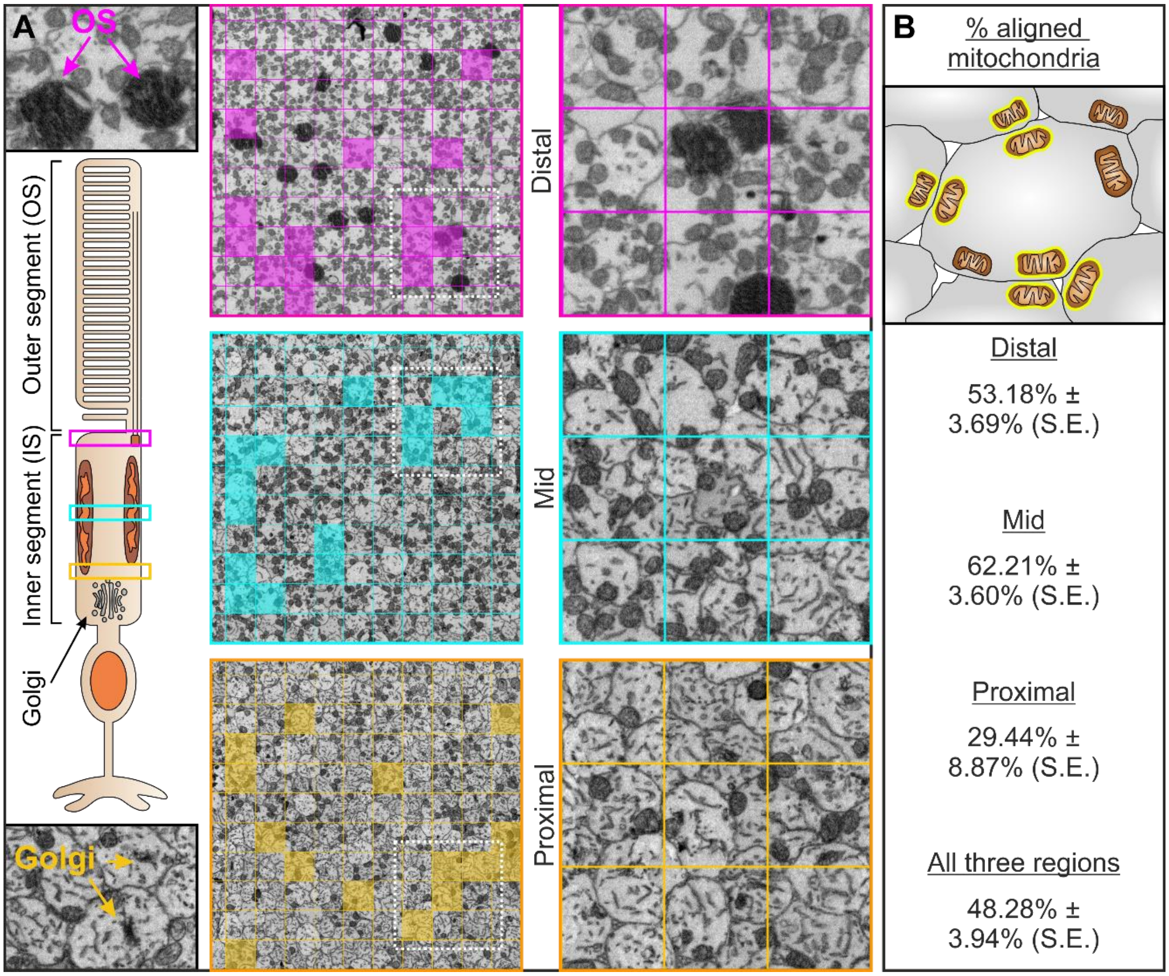

Fig. S1. There is greater alignment of the mitochondria in the middle and distal compared or proximal tip of the inner segment when viewing SBFSEM slices. A distal SBFSEM slice (purple) was identified due to the presence of outer segments (OS) whereas a proximal slice (yellow) the Golgi are visible. A mid-region (cyan) was picked half-way between distal and proximal slices. To randomly assess the mitochondrial alignment between IS a grid was overlaid onto slices and a random number generator used to pick the 15 regions. The greatest mitochondrial alignment was found in the middle and distal regions of the inner segment. Measurements from 146 mitochondria at the distal, 111 from the middle and 48 from the proximal region

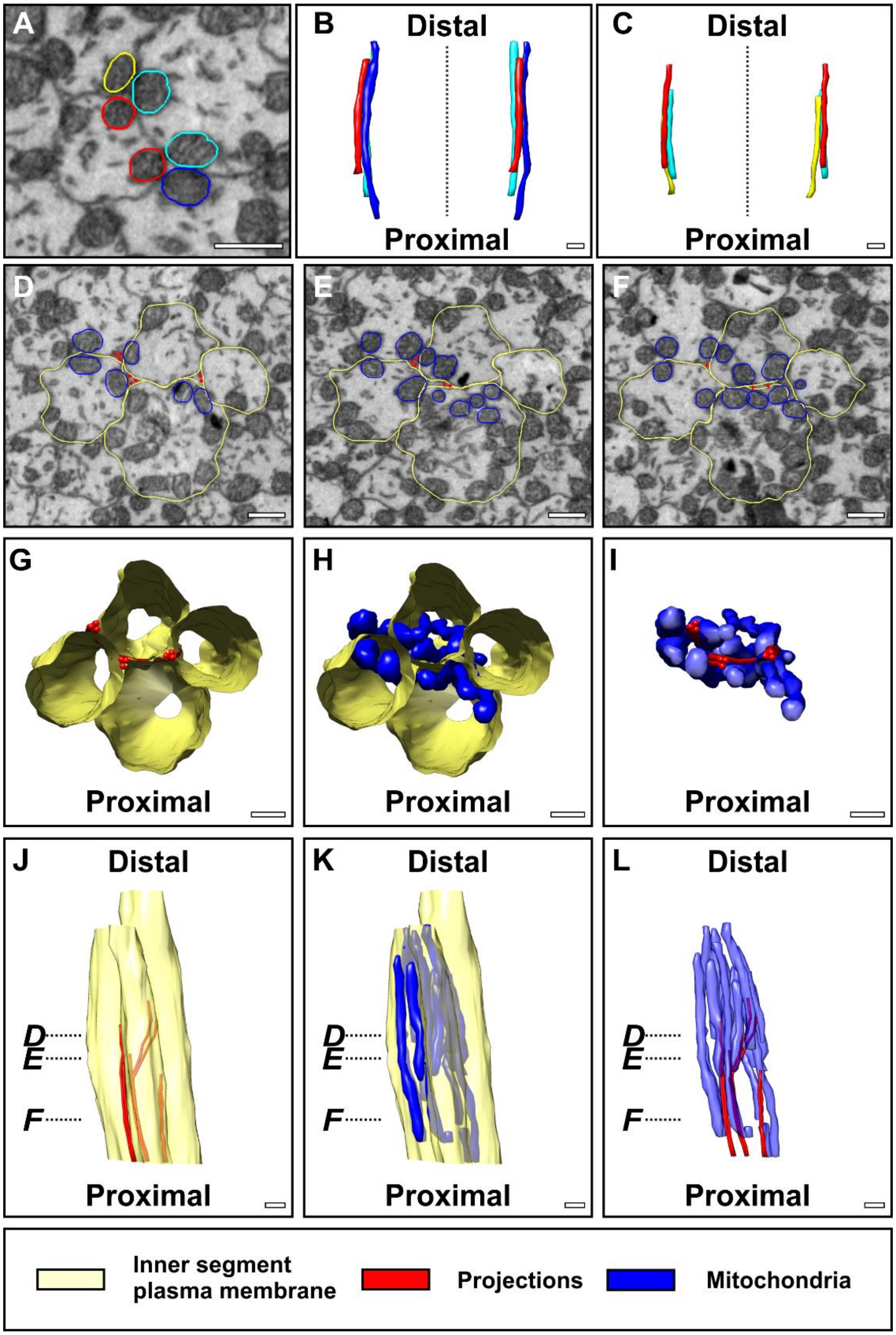

Fig. S2. Further examples of the mitochondrial alignment of neighbouring inner mitochondria as well as modelling of the projections seen in the extracellular space. (A-C) Segmentation of mitochondria from neighbouring inner segments and models generated showing them running side-by-side through the depth of the inner segment. (D-F) Segmentation of inner segment plasma membrane, mitochondria and projections. (G-L) The models generated from the segmentation show the projections are positioned close to mitochondria and run approximately halfway up the inner segment. Scale: 1µm

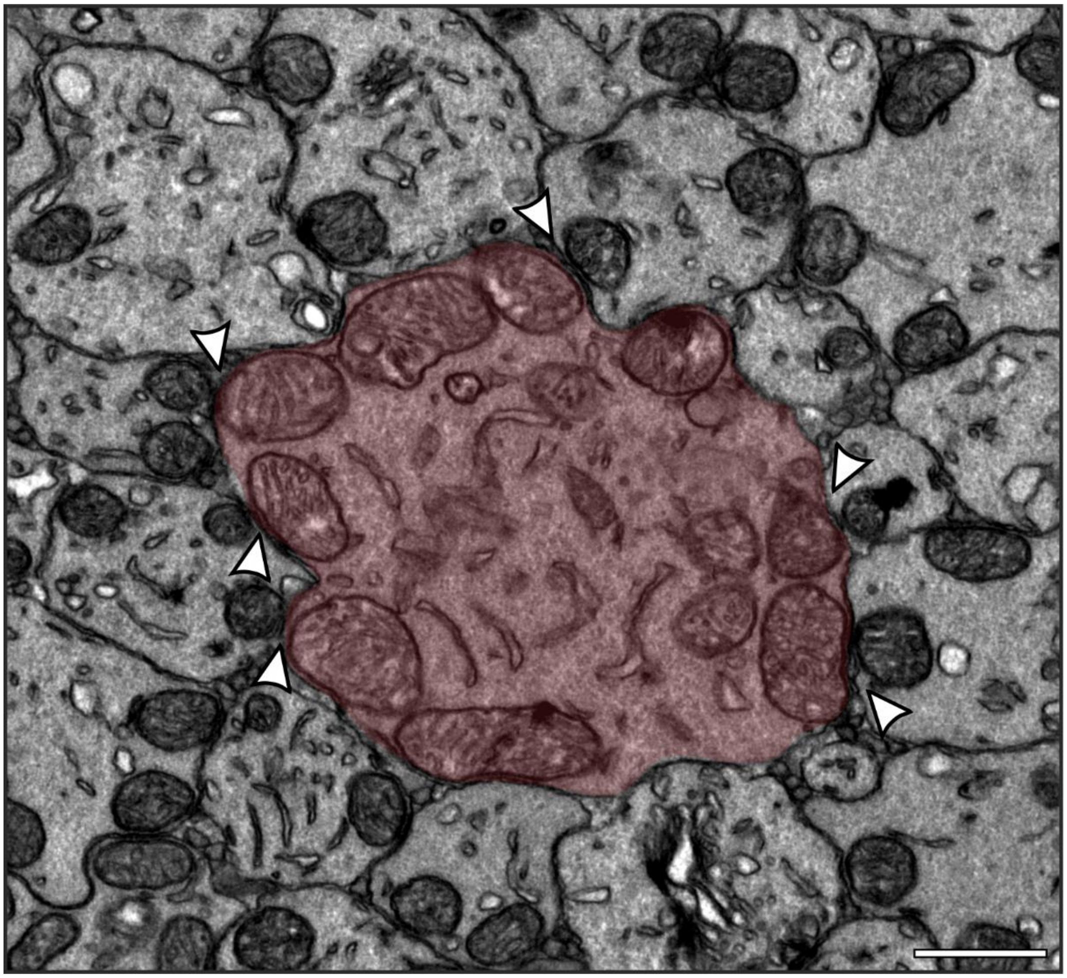

Fig. S3. Neighbouring photoreceptor mitochondrial alignment exists between cones and rods. The larger central cell (false coloured red) in the electron microscopy image is a cone that can be differentiated from rods due to its larger diameter and more electron lucent mitochondria. Alignment of mitochondria between cones and neighbouring rods indicated by the white arrowheads. Scale: 1µm

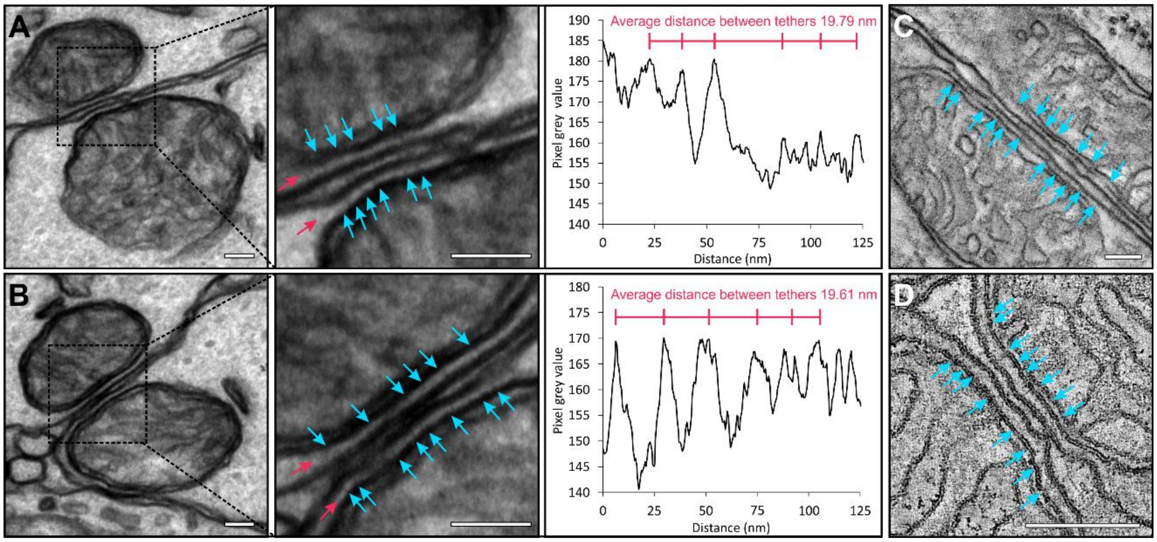

Fig. S4. Further images showing the tethering of mitochondria to the plasma membrane. (A & B) Electron microscopy images with high magnification images in the panels on the right and (C & D) slices from tomograms generated of inner segments. (A & B) Pixel intensity was measured along lines placed between the mitochondrial outer membrane and the plasma membrane as indicated by the red arrows. The results from the two lines drawn within each image were averaged together to generate plots in which the peaks represent the position and periodicity of the tethers. The blue arrows indicate the position of the tethers. Scale: (A – D) 100nm.

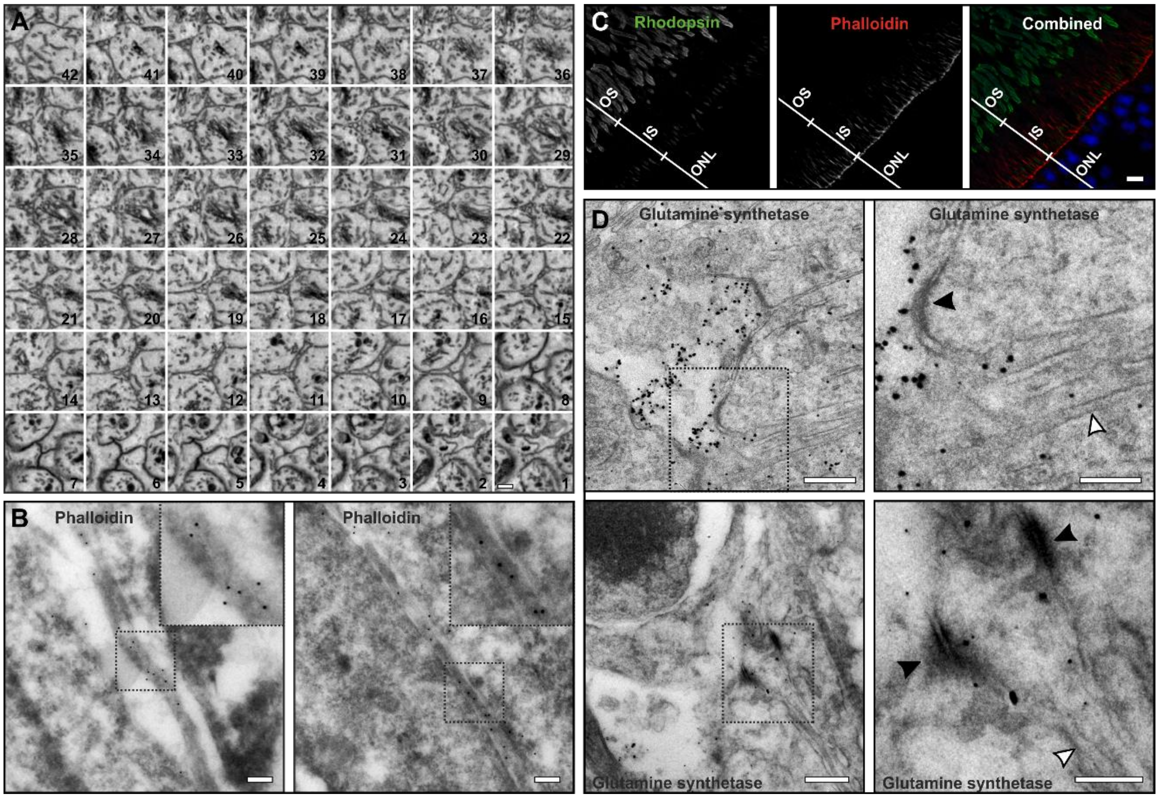

Fig. S5. Further evidence that the projections are actin enriched and originate from Müller glial cells. (A) SBFSEM images showing the projections opening up into the Müller glial cells surrounding the photoreceptors. (B) Immuno-EM of thawed frozen sections using phalloidin conjugated to biotin followed by anti-biotin labelling and 10nm protein-A-gold. The gold labelling localises to the projections indicating actin enrichment. (C) Immunofluorescence using an antibody against rhodopsin (green) as well as phalloidin (red) and DAPI (blue) staining. (D) Immuno-EM labelling of cryostat sections using antibody against the Müller glial cell marker glutamine synthetase. Scale: (A) 500nm, (B) 200nm, (C) 5µm, (D) left-handed panels 500nm and right-handed panels 250nm.

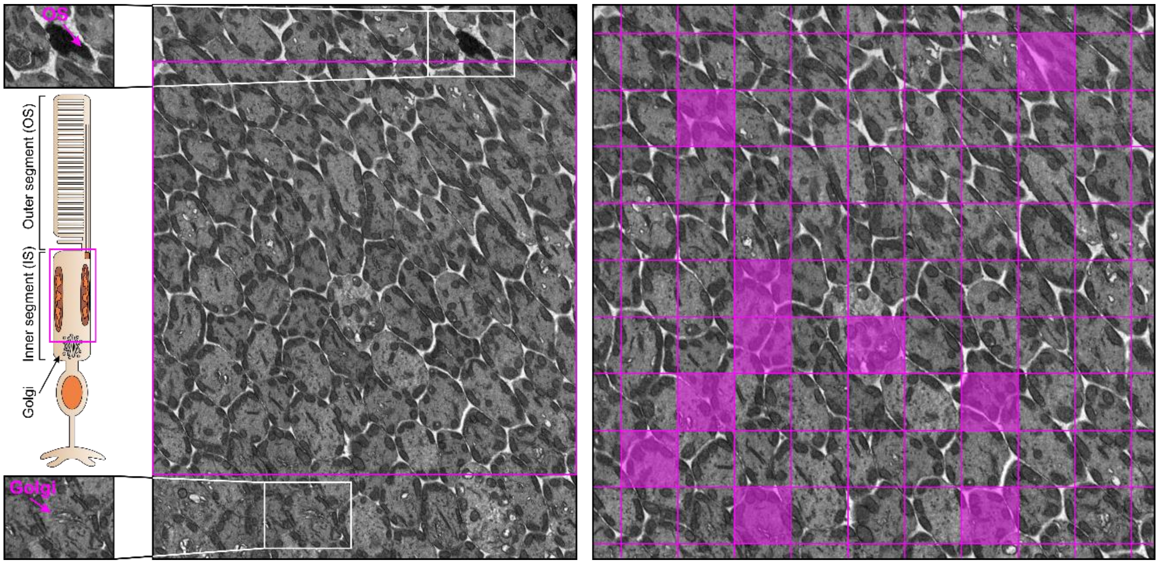

Fig. S6. The random selection of inner segments to assess mitochondria from a 6-month old mouse eye. There is a slight gradient of the orientation of the photoreceptors in the electron microscopy section, the top of the images contains distal portions of the inner segment as outer segments (OS) are visible, whereas the bottom of the image has proximal inner segments that contain Golgi. By examining inner segments between these two regions a grid can be overlaid and a random number generator used to pick boxes for assessment.

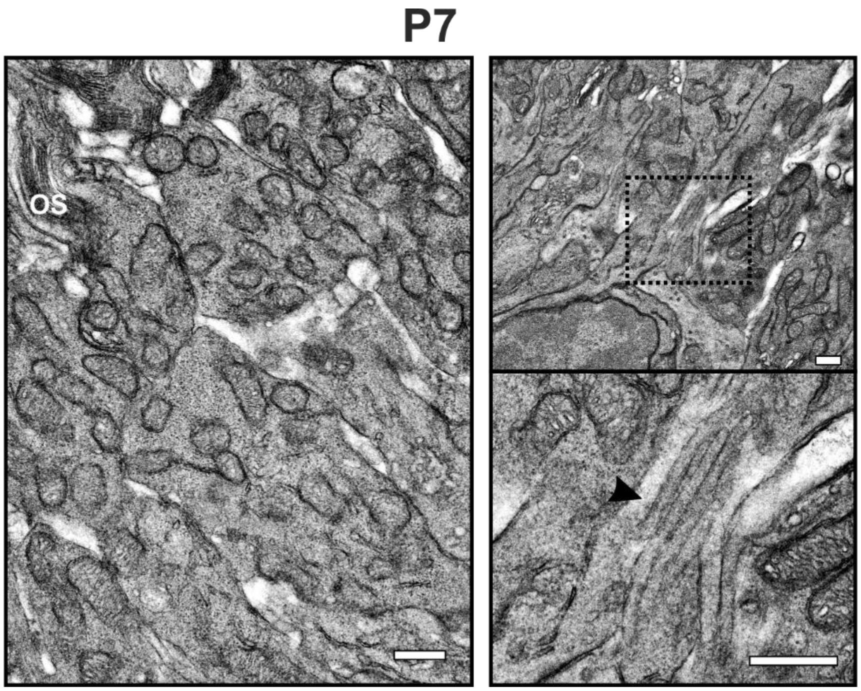

Fig. S7. At P7 there is presence of Müller glial cell projections between the immature photoreceptors as indicated by the black arrowhead. The photoreceptors at this time point are short and the outer segments (OS) are not fully developed. Scale: 500nm.

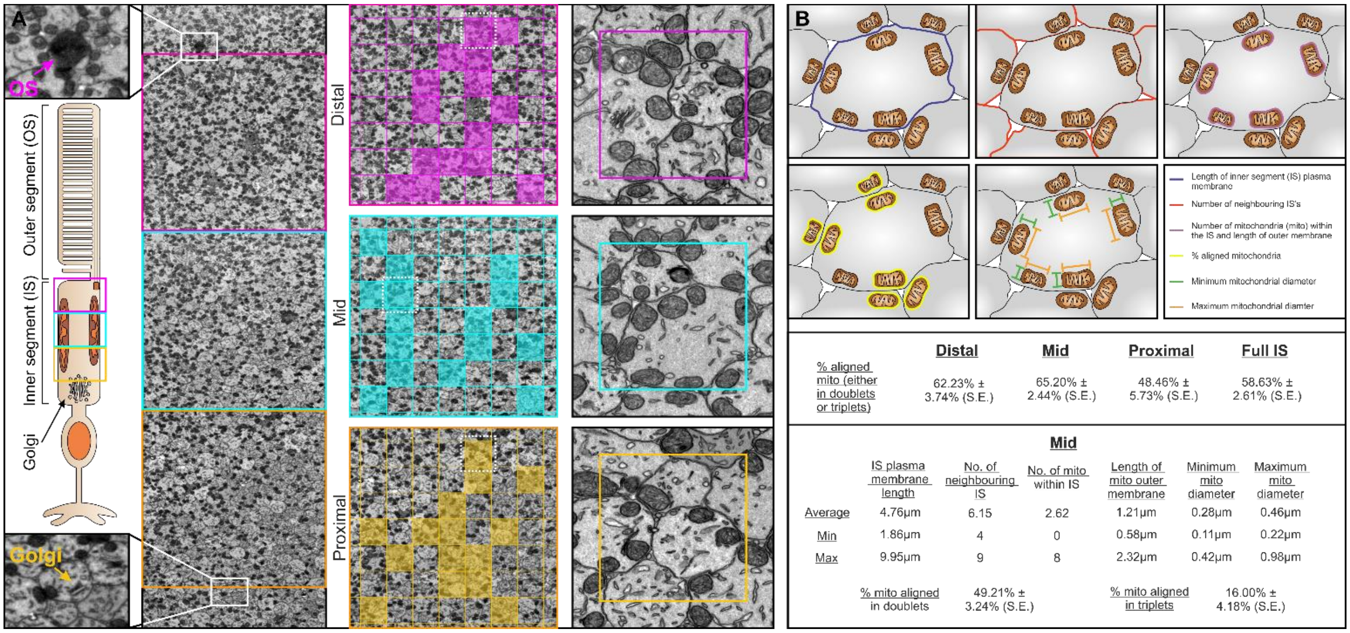

Fig. S8. There is greater alignment of neighbouring IS mitochondria within the mid IS region at P20 and quantitation provides important information regarding the inner segment and mitochondrial size and arrangement. (A) There is a slight gradient of the orientation of the photoreceptors as the outer segments are visible at the distal region of the IS at the top of the image and Golgi in the proximal region at the bottom of the image, therefore the image can be split into distal, mid and proximal regions. IS and mitochondria from random areas from these three regions were assessed. (B) The different types of measurements and results acquired from the random areas pick in (A) (taken from 237 mitochondria at the distal, 188 at the middle and 124 at the proximal regions). Note the proximal measurement is higher than that measured from the SBFSEM data in Fig. S1, which is due to the slight tilt in the orientation of the photoreceptors rather than a SBFSEM slice at the proximal IS respectively. The mid region of the IS had the greatest mitochondrial alignment between IS 65.20% ± 2.44% and this was further assessed to acquire measurements to generate a mathematical model (Fig. S9) to determine if the alignment of mitochondria is by chance.

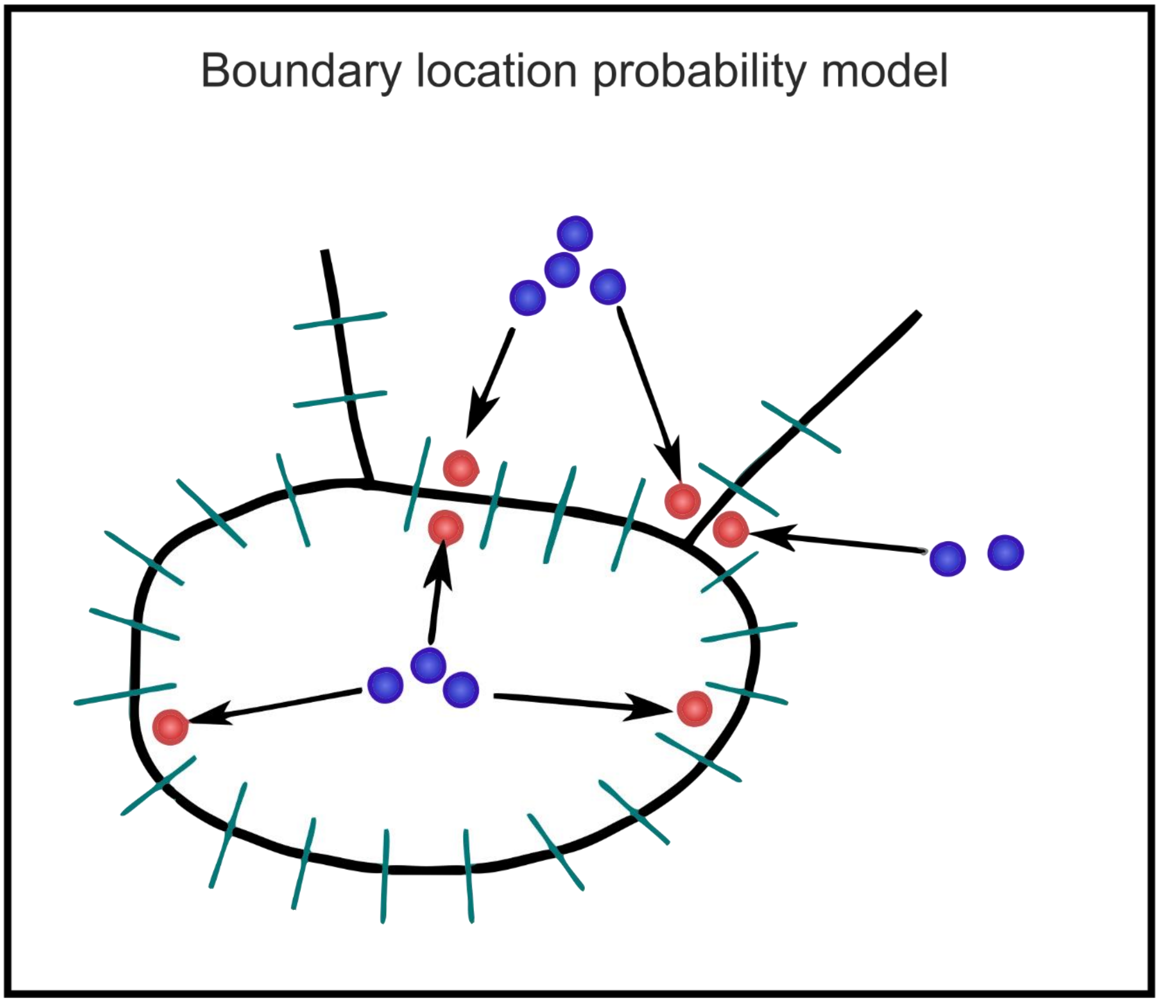

Fig. S9. Schematic diagram of the theoretical boundary location model used to calculate the alignment of neighbouring IS mitochondria when these are randomly placed against the plasma membrane. In this model mitochondria represented as circles (blue and red) are placed against the IS plasma membrane within random boundary perimeters separated by the length equivalent to the mitochondria diameter (red circles).

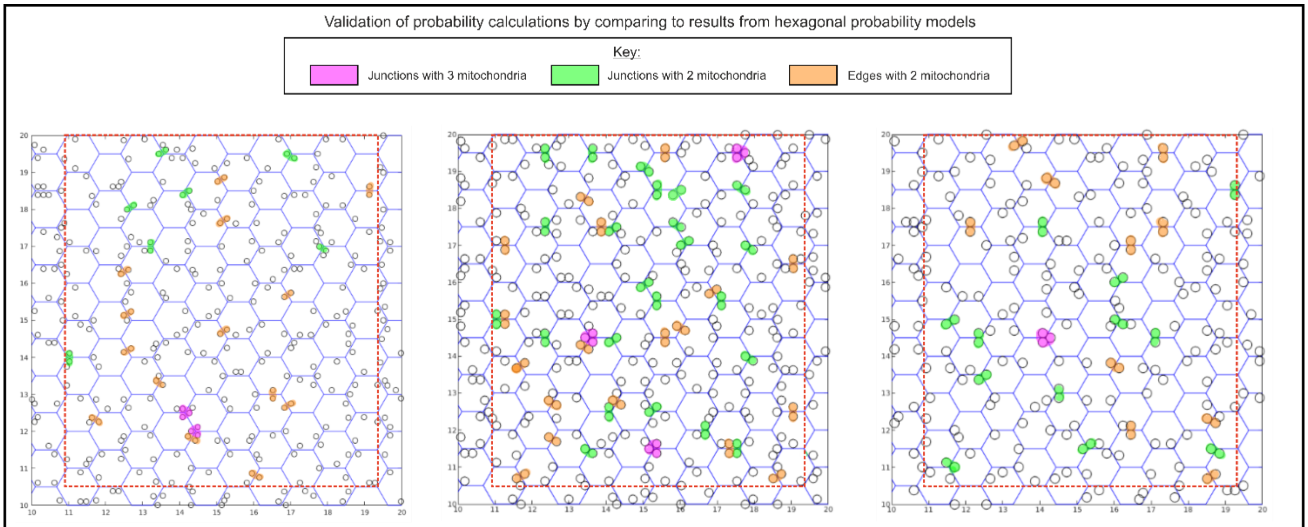

Grid size	10 by 10
Number of mitochondria per IS	3
Number of perimerter locations	18

	Results from hexagonal model	Expected Values from Probability Calculations
Number of junctions with 3 mitochondria	2	1
Number of junctions with 2 mitochondria	7	11
Number of edges with a single aligned pair	14	14
Number of edges with 2 or more aligned pairs	0	0
% mitochondria that are aligned	16.0%	17.8%
% mitochondria in pairs	14.0%	17.0%
% mitochondria in triplets	2.0%	0.8%

Grid size	10 by 10
Number of mitochondria per IS	3
Number of perimerter locations	12

	Results from hexagonal model	Expected Values from Probability Calculations
Number of junctions with 3 mitochondria	3	3
Number of junctions with 2 mitochondria	23	23
Number of edges with a single aligned pair	18	16
Number of edges with 2 or more aligned pairs	0	0
% mitochondria that are aligned	30.3%	28.6%
% mitochondria in pairs	27.3%	26.1%
% mitochondria in triplets	3.0%	2.5%

Grid size	10 by 10
Number of mitochondria per IS	2
Number of perimerter locations	12

	Results from hexagonal model	Expected Values from Probability Calculations
Number of junctions with 3 mitochondria	1	1
Number of junctions with 2 mitochondria	12	11
Number of edges with a single aligned pair	10	7
Number of edges with 2 or more aligned pairs	0	0
% mitochondria that are aligned	23.5%	19.6%
% mitochondria in pairs	22.0%	18.5%
% mitochondria in triplets	1.5%	1.1%

Fig. S10. The probability calculations were validated by comparing to the results obtained from a hexagonally tilted boundary location model generated in MATLAB. Agreement was observed between the two, validating the accuracy of the model equations (see supplementary methods).

A Measurements from electron microscopy images

IS Type	Iss with k mitos	Average no. of neighbouring photoreceptors	photoreceptor perimeter length (µm)	Frequency
0	0	5.38	3.04	8/65
1	1	5.77	4.02	13/65
2	2	6.17	4.60	18/65
3	3	5.90	4.82	10/65
4	4	6.75	5.51	4/65
5	5	6.75	6.11	4/65
6	6	7.00	6.59	6/65
7	7	8.00	7.03	2/65

B Idealised values for probability model

Mitochondria diameter set to 0.30 µm

IS Type	ISs with k mitos	No. of neighbouring photoreceptors	Edge size	Perimeter locations	Real location length (µm)	Idealised Perimeter Lengths using a location length set to 0.30 (µm)
0	0	5	1	10	0.30	2.8
1	1	6	1	12	0.33	3.36
2	2	6	2	18	0.26	5.04
3	3	6	2	18	0.27	5.04
4	4	6	2	18	0.31	5.04
5	5	7	2	21	0.29	5.88
6	6	7	2	21	0.31	5.88
7	7	8	2	24	0.29	6.72

Mitochondria diameter set to 0.45 µm

IS Type	ISs with k mitos	No. of neighbouring photoreceptors	Edge size	Perimeter locations	Real location length (µm)	Idealised Perimeter Lengths using a location length set to 0.45 (µm)
0	0	5	0	5	0.61	2.25
1	1	6	0	6	0.67	2.7
2	2	6	1	12	0.38	5.4
3	3	6	1	12	0.40	5.4
4	4	6	1	12	0.46	5.4
5	5	7	1	14	0.44	6.3
6	6	7	1	14	0.47	6.3
7	7	8	1	16	0.44	7.2

Mitochondria diameter set to 0.60 µm

IS Type	ISs with k mitos	No. of neighbouring photoreceptors	Edge size	Perimeter locations	Real location length (µm)	Idealised Perimeter Lengths using a location length set to 0.60 (µm)
0	0	5	0	5	0.61	3
1	1	6	0	6	0.67	3.6
2	2	6	0	6	0.77	3.6
3	3	6	0	6	0.80	3.6
4	4	6	0	6	0.92	3.6
5	5	7	1	12	0.51	7.2
6	6	7	1	12	0.55	7.2
7	7	8	1	14	0.50	8.4

C Comparison of mitochondrial alignment between mouse IS cross-sections and probability models

	Mouse IS cross-sections	Probability model predictions		
	Average mitochondrial diameter 0.46µm	Mitochondria diameter set to 0.30 µm	Mitochondria diameter set to 0.45 µm	Mitochondria diameter set to 0.60 µm
% aligned mitochondria	65.2%	16.3%	27.3%	44.3%
% mitochondria in pairs	49.2%	15.7%	25.0%	35.9%
% mitochondria in triplets	16.0%	0.6%	2.3%	8.4%

Fig. S11. Mitochondrial positioning probability models show lower expected mitochondrial alignment for randomly placed mitochondrial than that observed in real photoreceptor ISs. (A) Measurements taken from random locations of the P20 mouse mid IS and classed into different IS types depending on the number of mitochondria within the IS. (B) To test mitochondria of different diameters (30µm, 45µm and 60µm) in the probability model, the location length was set to these mitochondrial diameters and idealised perimeter lengths for the different IS types generated. (C) By comparing the probability model predictions against the real IS measurements (see Fig. S8.), the extent of expected random mitochondrial aligned to neighbouring IS mitochondria was found to be considerably lower that what was measured in the real IS. This indicates it is unlikely the mitochondrial arrangement in ISs is due to chance.

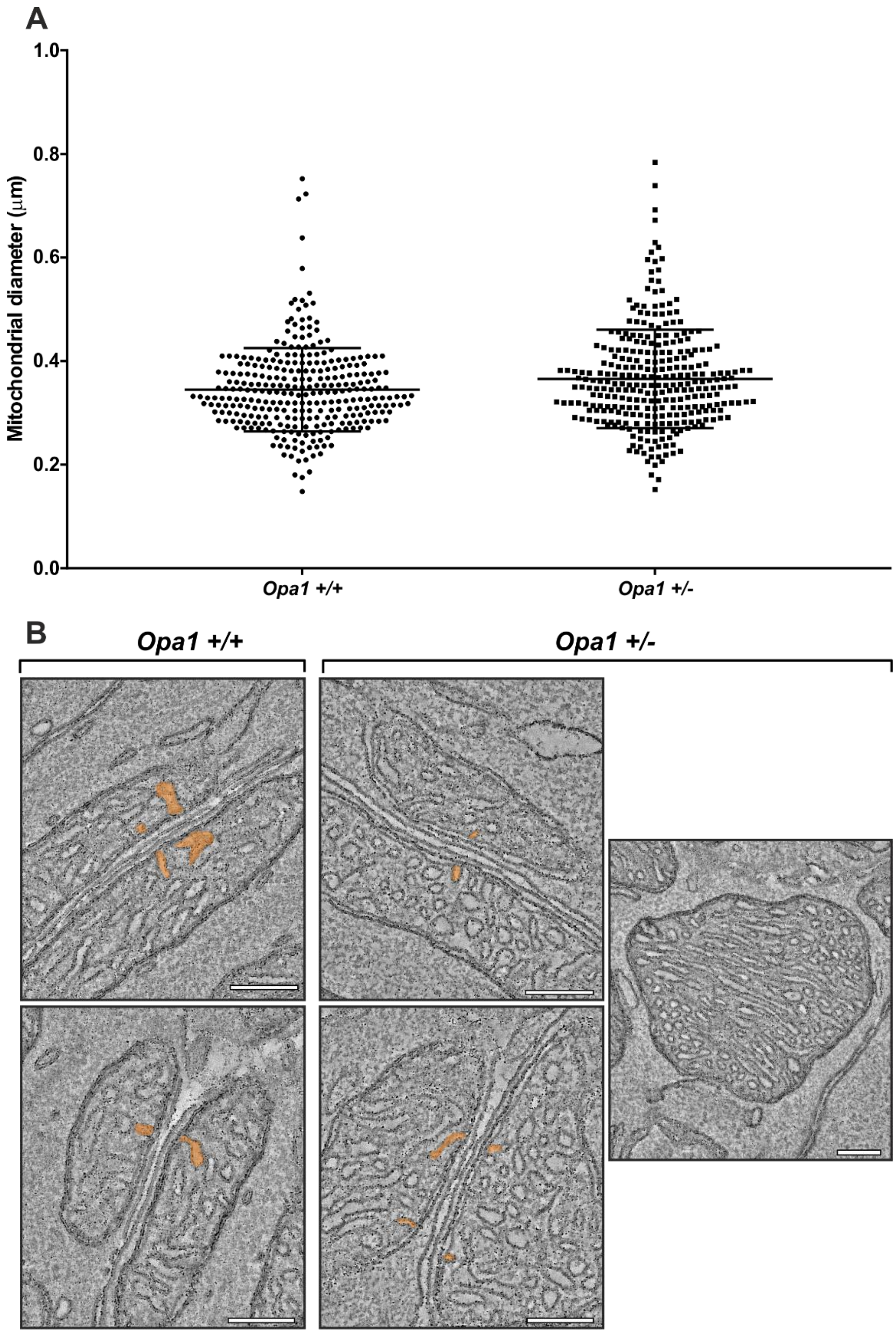

Fig. S12. Alternative representation of the mitochondria diameters measured and further tomograms showing evidence of cristae alignment in the Opa1 heterozygous knockout. (A) The distribution of 300 mitochondria diameters measured from the photoreceptor inner segments of 3 eyes from both *Opa1*$^{+/+}$ and *Opa1*$^{+/-}$ mice. (B) Further tomographic slices showing the existence of aligned cristae opening in the *Opa1*$^{+/+}$ and *Opa1*$^{+/-}$ mice and the far right-side panel show large mitochondria positioned away from the plasma membrane in the *Opa1*$^{+/-}$ mice have normal looking cristae. Scale: (B) 200nm.

Movie S1 (separate file). Model of selected mitochondria within three inner segments generated from SBFSEM data in Fig 1.

Movie S2 (separate file). Model of all mitochondria within three inner segments generated from SBFSEM data in Fig 1.

Movie S3 (separate file). Tomogram of mouse rod photoreceptor IS mitochondria from Fig. 2.

Movie S4 (separate file). Further tomogram of mouse rod photoreceptor inner segment mitochondria from Fig. 2.

Movie S5 (separate file). Model of mitochondrial inner and outer membranes and plasma membranes from two neighbouring photoreceptor inner segments.

SI References

1. T. Burgoyne, *et al.*, Rod disc renewal occurs by evagination of the ciliary plasma membrane that makes cadherin-based contacts with the inner segment. *Proc. Natl. Acad. Sci. U. S. A.* **112**, 15922–15927 (2015).

2. J. R. Kremer, D. N. Mastronarde, J. R. McIntosh, Computer visualization of three-dimensional image data using IMOD. *J. Struct. Biol.* **116**, 71–76 (1996).

3. J. W. Slot, H. J. Geuze, S. Gigengack, G. E. Lienhard, D. E. James, Immuno-localization of the insulin regulatable glucose transporter in brown adipose tissue of the rat. *J. Cell Biol.* **113**, 123–135 (1991).

4. T. Burgoyne, A. Lane, W. E. Laughlin, M. E. Cheetham, C. E. Futter, Correlative light and immuno-electron microscopy of retinal tissue cryostat sections. *PloS One* **13**, e0191048 (2018).

5. J. A. Rice, *Mathematical Statistics and Data Analysis (Third ed.)* (Duxbury Press, 2007).
